# The Therapeutic Pipeline for Eosinophilic Esophagitis: Current Landscape and Future Directions

**DOI:** 10.3390/ph18121882

**Published:** 2025-12-12

**Authors:** Andrea Pasta, Luisa Bertin, Amir Mari, Francesco Calabrese, Amir Farah, Giulia Navazzotti, Matteo Ghisa, Vincenzo Savarino, Edoardo Vincenzo Savarino, Edoardo Giovanni Giannini, Elisa Marabotto

**Affiliations:** 1 Gastroenterology Unit, Department of Internal Medicine, University of Genoa, 16132 Genoa, Italy; andrea.pasta@edu.unige.it (A.P.); vsavarin@unige.it (V.S.);; 2 Department of Surgery, Oncology and Gastroenterology, University of Padua, 35122 Padua, Italy; 3 Gastroenterology Unit, Azienda Ospedale Università di Padova, 35128 Padua, Italy; 4 Gastroenterology Unit, Nazareth EMMS Hospital, Nazareth 16100, Israel; 5 The Azrieli Faculty of Medicine, Bar Ilan University, Ramat Gan 15208, Israel; 6 IRCCS Policlinic San Martino Hospital, Viale Benedetto XV, 6, 16132 Genoa, Italy; 7 Department of Surgery, Medical College of Wisconsin, Milwaukee, WI 53226, USA

**Keywords:** eosinophilic esophagitis, therapeutic pipeline, biologics, type-2 inflammation, IL-4/IL-13 blockade, IL-5 blockade, JAK-STAT inhibitors, targeted drug delivery, precision medicine, dysphagia

## Abstract

Eosinophilic esophagitis (EoE) has emerged as a major cause of dysphagia and food impaction worldwide. This narrative review traces the evolving therapeutic pipeline for EoE, highlighting agents spanning from late-stage clinical development to final approval. We summarize mechanistic insights that have driven a shift from broad immunosuppression to precise inhibition of type-2 inflammatory pathways, including blockade of key interleukin pathways. Randomized trials have demonstrated histologic and symptomatic gains, yet regulatory approvals and optimal positioning within treatment algorithms are pending. Parallel innovations in drug delivery aim to maximize mucosal exposure while minimizing systemic burden. Key challenges include heterogeneity in disease phenotype, paucity of long-term safety data, and the need for non-invasive biomarkers to guide precision prescribing. Cost considerations and patient preferences will shape adoption. By integrating advances across immunology, formulation science and clinical trial design, the therapeutic pipeline for EoE holds promise to transform care from empirical suppression to mechanism-based disease modification.

## 1. Introduction

Eosinophilic esophagitis (EoE) is a chronic, immune-mediated inflammatory disease of the esophagus characterized by esophageal dysfunction and eosinophilic infiltration of the mucosa in the absence of other causes of eosinophilia [1]. Since its identification in the early 1990s, advancements in diagnostic techniques, especially using endoscopy with biopsy, have drastically improved its recognition and management [1,2,3,4].

The etiology of EoE is complex, involving a combination of genetic predisposition, such as variants in *CAPN14* and *TSLP* genes, immune dysregulation, alterations in the gut microbiome, environmental influences, and dietary triggers [5,6]. The disease is primarily driven by a type 2 helper T-cell (Th2) immune response, which leads to chronic inflammation, tissue remodeling, esophageal fibrosis and loss of esophageal function [1,7,8,9,10,11]. From a mechanistic point of view, type-2 inflammatory pathways set up a coordinated sequence culminating in tissue eosinophilia, fibroblast activation, and progressive esophageal remodeling [12]. IL-4 and IL-13 upregulate epithelial eotaxin-3 (CCL26), establishing a potent chemotactic gradient that sustains eosinophil recruitment and retention within the mucosa. IL-5 further promotes eosinophil maturation, survival, and degranulation, amplifying cytotoxic injury to the epithelium [13]. Persistent exposure to these cytokines activates TGF-β–dependent fibroblast signaling, driving extracellular matrix deposition, subepithelial fibrosis, and reduced esophageal compliance. This interplay between epithelial injury, eosinophil-mediated inflammation, and TGF-β–driven remodeling underlies the transition from inflammatory to fibrostenotic disease, thereby linking upstream immune activation with the clinical phenotype [14]. Given its immune-mediated nature, EoE frequently coexists with other atopic conditions, including asthma, allergic rhinitis, and atopic dermatitis, further complicating disease management [15,16].

The prevalence of EoE has increased substantially over the past few decades, largely due to heightened clinical awareness and improved diagnostic criteria [5]. In a 2023 systematic review covering 1976–2022, the authors estimated that the global incidence of EoE was approximately 5.31 cases per 100,000 people per year, with a prevalence of 40.04 cases per 100,000 individuals [17]. However, more recent data indicate a substantial increase in prevalence, reaching up to 74.4 cases per 100,000 between 2017 and 2022 [17]. In the United States, the estimated prevalence in 2022 rose to approximately 142 cases per 100,000, with an incidence of around 34 cases per 100,000 person-years [18]. The disease is predominantly diagnosed in males, with a male-to-female ratio of approximately 3:1, and is most commonly detected in children and young adults, and higher prevalence rates are reported in Western countries [5,19].

The management of EoE has evolved considerably over the years, moving from empirical treatments toward a more personalized, evidence-based approach [20]. Current therapeutic strategies focus on eliminating dietary triggers, reducing inflammation with pharmacologic agents, and managing complications through endoscopic interventions [7,20,21,22,23,24,25,26,27].

Several dietary approaches have been investigated, with the six-food elimination diet (SFED) being the most restrictive but also one of the most effective, achieving histologic remission in many patients by eliminating the six most common food allergens: milk, wheat, eggs, soy, nuts, and seafood [28]. However, adherence to such a strict regimen can be challenging. Less restrictive diets, such as the four-food elimination diet (FFED), which excludes dairy, wheat, eggs, and soy, offer a more feasible approach with slightly lower but still significant efficacy rates [29,30]. The one-food elimination diet (OFED), which typically removes cow’s milk alone, has been shown to induce remission in approximately 50–55% of cases, making it an attractive option for patients seeking a simpler dietary intervention [31,32]. The most restrictive dietary approach is the elemental diet, which relies on amino acid–based formulas to completely eliminate all potential allergens, has demonstrated the highest efficacy rates of up to 71–72%, but its practicality is often limited due to cost and poor palatability [33].

Proton pump inhibitors (PPIs) are well-recognized for their anti-secretory and anti-inflammatory effects [34], achieving remission in approximately 50% of patients by inhibiting IL-13-induced eotaxin-3 production and reducing eosinophilic recruitment [1,7,35,36,37]. Topical steroids have demonstrated histologic remission rates exceeding 80%, making them a highly effective therapy [38,39]. More recently, biologic therapies have emerged as a promising treatment option. While dietary modifications, PPIs, and corticosteroids remain first-line treatments, the introduction of biologic agents represents a paradigm shift in EoE management, particularly for patients with refractory disease [38,40]. For patients with fibro-stenotic complications, endoscopic dilation serves as an effective intervention to relieve esophageal strictures and improve swallowing [41].

Despite major advances in understanding EoE and refining first-line therapy, clinicians lack an up-to-date, integrated overview of the fast-evolving treatment pipeline [12]. Earlier evidence has played a key role in defining current management, but much of it predates the latest approvals and late-phase trial readouts for topical steroids, biologics, and small molecules. Building on this existing literature, the present review aims to fill this gap by providing an updated, mechanism-based synthesis of the main agents now approved or in development—from next-generation swallowed steroids to novel small molecules—critically evaluating their efficacy, safety, and durability.

## 2. Pharmacotherapies for Eosinophilic Esophagitis

The management of EoE has traditionally followed the “four Ds” paradigm: Drugs (swallowed corticosteroids or proton-pump inhibitors), Dietary elimination, esophageal Dilation for strictures, and attention to Disease-associated psychosocial factors [42,43]. This scenario is now changing, as improved understanding of EoE’s Th2 (type 2) inflammatory pathways has spurred development of targeted therapies [44]. Numerous drugs, ranging from topical steroids to biologic monoclonal antibodies, have been or are being evaluated for EoE, and in this review, we aim to examine the principal mechanisms in depth throughout the text.

**Figure 1:**
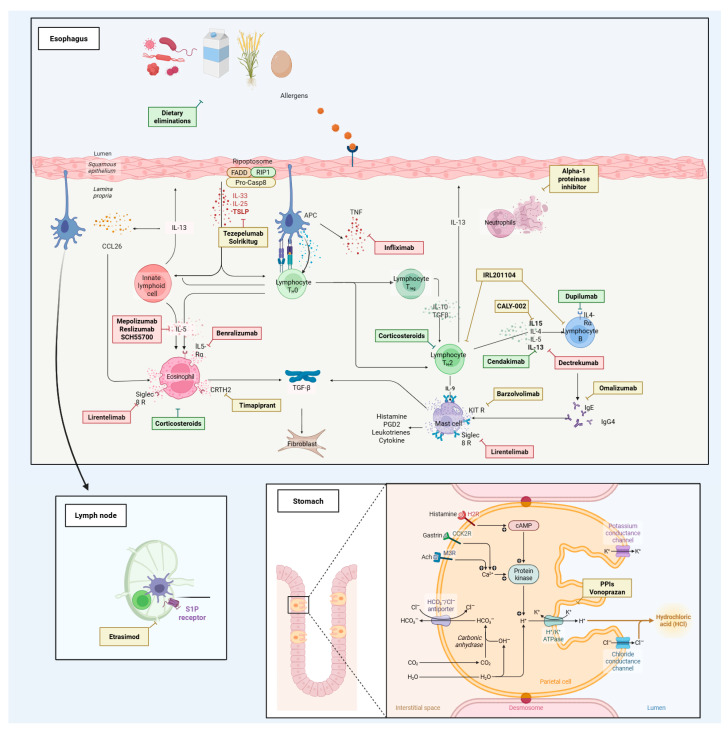
Pathogenic pathways in eosinophilic esophagitis (EoE) and points of therapeutic intervention. Schematic of the esophageal mucosa highlighting how food and aeroallergens traverse an impaired epithelial barrier to initiate type-2 inflammation. Epithelial-derived cytokines (TSLP, IL-33, IL-25) prime dendritic cells and innate lymphoid cells and promote Th2 polarization, with downstream production of IL-4/IL-13 and IL-5. IL-13 stimulates epithelial eotaxin-3/CCL26 and fibroblast activation (TGF-β–mediated remodeling), while IL-5 supports eosinophil maturation, recruitment, and survival. Mast cells amplify inflammation through mediators (histamine, PGD_2_, leukotrienes). Therapeutics are positioned at their site of action: dietary elimination removes upstream triggers; swallowed topical corticosteroids exert broad anti-inflammatory effects. Targeted agents include anti-TSLP (tezepelumab, solrikitug), anti-IL-4/IL-13 axis (dupilumab; anti-IL-13 agents cendakimab, dectrekumab, IRL201104), anti-IL-5/IL-5R (mepolizumab, reslizumab, benralizumab, SCH55700), anti-Siglec-8 (lirentelimab) and anti-KIT (barzolvolimab) for mast-cell pathways, anti-IgE (omalizumab), and anti-TNF (infliximab). CRTH2/DP2 antagonism (timapiprant) blocks PGD_2_-driven Th2/eosinophil trafficking. An inset shows lymph-node S1P receptor modulation (etrasimod) to limit lymphocyte recirculation. The gastric inset depicts acid-secretion pathways targeted by PPIs and the potassium-competitive acid blocker vonoprazan. Additional exploratory approaches (e.g., alpha-1 proteinase inhibitor) are shown at putative sites of action. Arrows indicate stimulatory interactions; bars denote pharmacologic blockade.

**Figure 2:**
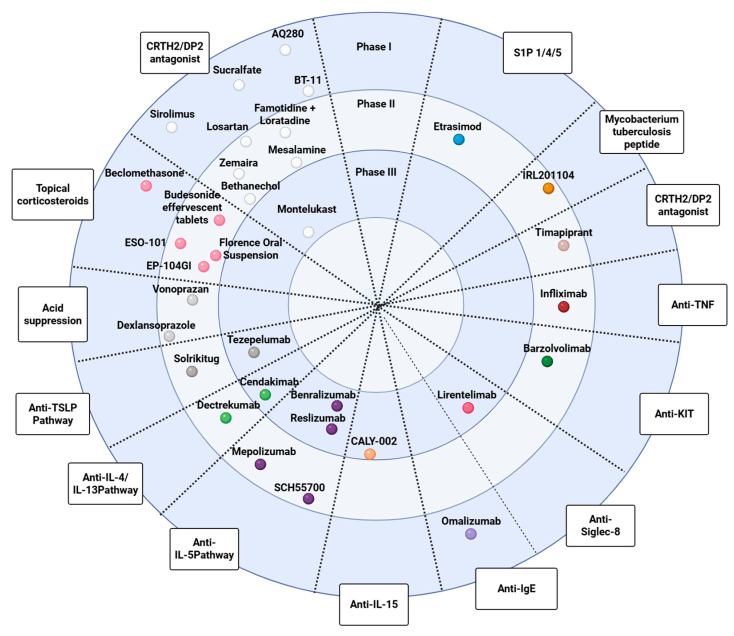
Therapeutic pipeline for eosinophilic esophagitis (EoE) organized by mechanism of action and stage of development. Radial “disc” schematic in which each spoke represents a pharmacologic class (i.e., topical corticosteroids, acid suppression, anti-TSLP, anti-IL-4/IL-13, anti-IL-5, anti-IL-15, anti-IgE, anti-Siglec-8, anti-KIT/mast cell, anti-TNF, CRTH2/DP2 antagonists, S1P modulators, and other emerging approaches). Concentric rings denote clinical-trial stage, from Phase I at the outer ring to Phase III toward the center. Individual dots indicate representative agents positioned by their predominant mechanism and the most advanced reported stage.

Table 1 summarizes phase 1 and early-phase studies investigating a broad spectrum of candidate therapies and mechanistic interventions for eosinophilic esophagitis and related eosinophil-associated gastrointestinal disorders. None of these agents has yet received a specific indication, and most trials primarily focused on pharmacokinetics, safety, bioavailability, and formulation aspects rather than definitive efficacy endpoints. Figure 1 maps key pathogenic pathways—epithelial barrier dysfunction, antigen presentation, Th2 polarization, eosinophil recruitment/activation, and mast-cell signaling—and overlays representative therapies at their points of action, illustrating how mechanistic insights are being translated into targeted treatment strategies. Complementing this picture, Figure 2 summarizes this therapeutic landscape, arranging agents by mechanism along radial axes and by development stage across concentric rings (from phase I to phase III), and positioning established options (PPIs, swallowed topical corticosteroids) alongside targeted classes such as anti-IL-4/IL-13, anti-IL-5, anti-TSLP, anti-Siglec-8, anti-KIT/mast-cell, anti-IgE, anti-TNF, and CRTH2/DP2 or S1P modulators.

### 2.1. Topical Swallowed Corticosteroids

Swallowed topical corticosteroids have been the mainstay of EoE therapy, working by locally suppressing esophageal inflammation. Fluticasone propionate swallowed via inhaler and oral budesonide suspension have long been used off-label [45,46]. To optimize delivery to esophageal tissue, novel formulations have been developed and tested in large Phase 2/3 trials, leading to formal approvals in some regions. From a formulation standpoint, these esophagus-targeted products are designed to prolong mucosal exposure time and luminal contact with inflamed tissue. Orodispersible tablets and viscous oral suspensions increase local steroid exposure along the esophageal mucosa, which likely contributes to the high histologic remission rates reported [47]. Swallowed budesonide formulations, effervescent tablets, and esophageal-retentive gels are specifically engineered to maximize esophageal residence time and achieve a more homogeneous coating of the mucosa, thereby aligning local delivery with mucosal pharmacokinetic and tissue deposition models that favor high topical drug concentrations at the site of inflammation, steep luminal–tissue gradients, and limited systemic absorption [48]. From a pharmacokinetic perspective, frequency and duration of administration remain key determinants of adherence to topical and systemic therapies for EoE. Currently available swallowed topical steroids have rapid gastrointestinal absorption, meaning short Tmax, and relatively short systemic half-lives, which limits systemic exposure but generally requires once- or twice-daily dosing to maintain adequate esophageal exposure. Emerging mucoadhesive and device-assisted formulations are specifically designed to decouple esophageal residence time from systemic half-life, prolonging local tissue contact despite rapid systemic clearance and thereby potentially enabling once-daily or even less-frequent dosing [48,49].

- **Budesonide Orally Disintegrating Tablets (ODT):** This formulation dissolves in saliva and coats the esophagus. In a Phase 3 placebo-controlled induction trial (6-week, EOS1 study), budesonide ODT 1 mg twice daily achieved complete clinical-histologic remission in 58% of adults versus 0% on placebo (primary endpoint). Histologic remission alone (≤5 eos/hpf) was reached in 93% on budesonide vs. 0% placebo [50]. A 48-week maintenance trial (EOS2) in patients who achieved remission showed 75% of patients on budesonide ODT (0.5 mg or 1.0 mg BID) remained in remission, compared to only 4% on placebo (*p* < 0.001). Time to relapse in the placebo arm was short (~87 days), underscoring the need for maintenance therapy in this chronic disease [51]. Budesonide ODT was well tolerated, with candidiasis occurring in ~12–16% on drug vs. none on placebo, and a few asymptomatic cortisol decreases were noted [51]. Budesonide ODT became the first esophagus-targeted therapy approved for EoE, authorized in Europe in 2018 for adults [52] and subsequently in Canada and Australia [53], but it is still not approved by the US Food and Drug Administration (USFDA). Consistently, a recent 11-center prospective real-world cohort (*n* = 233) using budesonide ODT 1 mg BID for 12 weeks (with most patients tapered to 1 mg QD for maintenance) showed deep histologic remission in 84% at induction and maintenance of deep remission in 78% at 52 weeks, with mainly mild adverse events (12% overall) and oral candidiasis in 9% [54].
- **Budesonide Oral Suspension (BOS):** This approach is a viscous budesonide slurry that the patient swallows to coat the esophagus. In the 12-week induction Phase 3 U.S. trial, which included 318 pediatric and adult patients, BOS 2 mg twice daily significantly outperformed placebo, with 53.1% vs. 1.0% of patients achieving histologic remission (≤6 eos/hpf; Δ ≈ 52%, *p* < 0.001) [55]. Symptomatic improvement (≥30% reduction in Dysphagia Symptom Questionnaire score) occurred in 52.6% on BOS vs. 39.1% on placebo (Δ13%, *p* = 0.024). Endoscopic severity scores (EREFS) and mean dysphagia scores also improved significantly more with BOS [55]. The suspension was generally safe and well-tolerated, with mostly mild-to-moderate adverse events (similar to placebo) [55]. Based on these results, budesonide BOS was approved by the USFDA in early 2024 as the first official oral treatment for EoE in the US [56]. The approval is for induction therapy (up to 12 weeks) in adolescents (≥11 years) and adults with active EoE. Notably, maintenance use beyond 12 weeks is not yet USFDA-approved [53], although long-term extension studies on other formulations show continued efficacy and safety up to at least 48 weeks [51].
- **Fluticasone Orally Disintegrating Tablet (APT-1011):** Fluticasone, a potent topical steroid, was originally used by swallowing the puff from an asthma inhaler, with variable success [45]. To improve delivery, an effervescent oral disintegrating tablet (APT-1011) was developed. The Phase 2b FLUTE trial evaluated APT-1011 in 106 adults over 12 weeks of induction and 40 weeks of maintenance [57]. At week 12, histologic remission (≤6 eos/hpf) was achieved in 80–86% of patients on high-dose regimens (e.g., 3 mg BID or 1.5 mg BID) versus 0% on placebo (*p* < 0.001). Endoscopic severity scores markedly improved on fluticasone with EREFS falling from 5 to 2 points, while barely changing on placebo. Dysphagia symptom frequency also improved significantly more with fluticasone than placebo, and improvements were sustained through 52 weeks on continued therapy [57]. The BID regimens had more frequent candida infections, but overall, APT-1011 was well tolerated. Notably, even a lower dose, 3 mg once daily at bedtime, achieved a 67% remission rate with a favorable safety profile [57]. To date, a Phase 3, randomized, double-blind study (FLUTE 3) has enrolled adults with eosinophilic esophagitis between December 2022 and August 2024. Participants received a 3 mg bedtime orally disintegrating tablet of APT-1011 for 24 weeks. Investigators have evaluated dysphagia symptom reduction, histologic remission, and safety. Completers may have entered a longer open-label extension, allowing efficacy monitoring. Secondary outcomes included quality-of-life scores and corticosteroid exposure over time [58].
- **Extended-release fluticasone suspension (EP-104GI):** In an open-label, dose-escalation, phase 1b/2a study (RESOLVE), adults with active EoE received a single intra-esophageal injection of polymer-coated fluticasone crystals at 4–20 sites (4–64 mg). Among 18 patients treated with 6 mg, the peak eosinophil counts fell 55–94%, and the highest 64 mg dose achieved 62% histologic remission (≤6 eos/hpf) plus 65–66% reductions in composite EoEHSS stage/grade. Straumann Dysphagia Index scores dropped 3–5 points (46–71%) by week 12 and remained improved at week 24–36 in earlier cohorts. Serum cortisol/glucose were normal, and only mild–moderate procedure-related AEs (e.g., transient chest pain, nausea) occurred—no oral/GI candidiasis or adrenal suppression [59].
- **Mometasone furoate (ESO-101):** ESO-101 is a thin, muco-adhesive polymer film that unrolls in the esophagus at bedtime, providing prolonged, targeted steroid exposure while maintaining <0.1% systemic bioavailability. In the ACESO Phase 2 randomized, double-blind induction study (28 days; *n* = 43), a once-nightly 800 µg dose lowered peak eosinophil counts by a mean 49 eos/hpf versus +7 with placebo (*p* = 0.03). Histologic remission (<15 eos/hpf) was reached in 48% of treated adults and deep remission (<6 eos/hpf) in 44%, compared with 0% on placebo; endoscopic inflammatory + fibrostenotic scores (EREFS) likewise improved (median −3 vs. +1). No treatment-emergent serious adverse events, candidiasis, or clinically significant cortisol changes were observed, supporting an excellent safety profile [60]. Building on these results, ESO-101 is now being evaluated in an ongoing global Phase 3 trial (NCT04849390) designed to confirm efficacy and support regulatory filing [61]. Earlier evidence with a mometasone furoate oral spray (200 µg four times daily for 8 weeks) showed a median 6.5-point reduction in the Watson Dysphagia Scale versus 0 with placebo (*p* < 0.05), demonstrating symptomatic benefit even without esophageal-specific delivery [62].
- **Florence Oral Suspension (FOS):** This swallow-and-coat liquid formulation is being tested as another esophagus-targeted topical steroid for EoE. A national, randomized, double-blind Phase 2 study in Brazil (EMS0718; NCT02873468) is currently enrolling 116 adults to compare three twice-daily concentrations—30, 60 and 90 µg/mL—with placebo over a 100-day induction period [63]. The primary endpoint is complete histologic response (≤6 eosinophils/high-power-field) at day 100, with parallel assessments of symptom relief and safety. Recruitment began on 19 April 2021 and remains underway; topline efficacy and safety results are still pending, and study completion is projected for 2026 [63]. If the dose-ranging data confirm meaningful remission with an acceptable safety profile, FOS could advance to Phase 3 trials and expand the therapeutic toolkit for long-term management of this chronic disease. At present, it remains an investigational product with no regulatory approvals. Additionally, early Phase 1b/2a data from the RESOLVE study of EP-104GI (extended-release fluticasone via intra-esophageal injection) reported in late September 2025 showed that the highest-dose cohort (8 mg per injection) achieved the largest improvements to date in histologic/tissue outcomes and eosinophil reduction with no serious adverse events or candidiasis; based on these results, the 8 mg dose was endorsed for testing in the randomized Phase 2b and the trial size is being expanded to ≥120 patients [64].

In summary, topical corticosteroids have demonstrated high efficacy in inducing histologic remission and improving symptoms in EoE, with a favorable safety profile, with localized candida infections reported as the most frequent adverse event [50,51,55,57]. Table 2 summarizes phase 2 and 3 clinical trials of topical corticosteroids and related steroid-based formulations for eosinophilic esophagitis.

### 2.2. Potassium-Competitive Acid Blocker

In eosinophilic esophagitis (EoE), bursts of refluxed acid flip open epithelial TRPV1 proton sensors, launching an ATP-fuelled NF-κB loop that up-regulates eotaxin-3, weakens tight junctions and locks in eosinophil-driven type-2 inflammation [65]. Damping that upstream acid signal has long been the rationale for high-dose PPIs, which irreversibly alkylate the gastric H^+^/K^+^-ATPase after acid-dependent activation. Vonoprazan strikes the same enzyme but by a different mechanism: it settles in the luminal K^+^ pocket as a reversible, potassium-competitive acid blocker (P-CAB), achieving sub-nanomolar affinity, meal-independent onset and freedom from CYP2C19 variability [66,67]. In a 118-patient Japanese study, 8 weeks of vonoprazan 20 mg daily led to complete symptom relief in ~76% of patients, with similarly high rates of endoscopic and histologic remission; outcomes did not significantly differ from those achieved with standard or high-dose PPI regimens [68]. In the same direction, a more recent multicenter series (*n* = 236) reported symptomatic, endoscopic, and histological response rates of approximately 80–87% after PPI/P-CAB therapy, although complete normalization of all three parameters was rare, with ~8% of patients attaining simultaneous symptomatic, endoscopic, and histological remission [69]. The durability of response is now being tested prospectively: a randomized, double-blind, placebo-controlled Phase 2 study in adults and adolescents (NCT06851559) will compare vonoprazan 10 mg and 20 mg once daily with placebo over a 12-week induction phase and assess maintenance to 52 weeks, with co-primary endpoints of symptomatic improvement and histologic remission [70]. In terms of safety, vonoprazan’s profile appears similar to that of PPIs. Short-term trials in EoE have not revealed unique adverse effects, and in long-term studies for acid-related disorders, vonoprazan demonstrated comparable overall adverse event rates to PPI therapy [67].

### 2.3. Biologic Therapies Targeting Type 2 Inflammation

EoE is driven by a Th2-skewed allergic cascade dominated by IL-5, IL-13, and IL-4, which coordinate eosinophil trafficking, activation, and survival. Upstream epithelial cytokines like thymic stromal lymphopoietin (TSLP) and IL-33 amplify this loop, while eotaxin-3 (CCL26) provides a potent chemokine gradient. Chronic signaling promotes tissue remodeling via TGF-β–mediated fibrosis. These insights have spurred trials of targeted biologics originally successful in asthma or atopic dermatitis and now translated in EoE [12,71]. Therapeutic strategies in EoE range from broadly acting anti-inflammatory drugs to highly selective biologics and small molecules [72]. Whereas broad agents modulate multiple pathways and may require less tissue specificity and a higher risk of systemic adverse effects over time, refined pathway-selective inhibition aims at focusing on disease-driving mechanisms in the esophagus, with the potential for better tissue specificity and fewer systemic safety concerns [12]. Table 3 provides an integrated overview of biologic and other targeted therapies investigated in eosinophilic esophagitis, grouped according to their predominant mechanistic pathway. Overall, dupilumab is currently the only biologic approved for EoE (US FDA 2022), while other leading agents remain in phase 3 development without formal regulatory approval.

#### 2.3.1. IL-5 Pathway Inhibitors (Anti-IL-5 and IL-5Rα)

IL-5 is the key eosinophil growth, activation, and survival factor. This cytokine acts as the master survival and activation signal for eosinophils. Engagement of its heterodimeric receptor triggers JAK–STAT, MAPK, and PI3K pathways, sustaining eosinophilic inflammation and tissue remodeling [12]. Monoclonal antibodies against IL-5 or its receptor (IL-5Rα) aim to deplete or disable eosinophils, thus addressing what was presumed to be the central effector cell in EoE. Several such biologics were tested in EoE, but results have been underwhelming in terms of symptom improvement, altering the initial hypothesis that simply removing eosinophils would cure EoE [73].

- **Mepolizumab (anti-IL-5):** Mepolizumab, an IgG1 antibody binding IL-5, was one of the first biologics tried in EoE. Nearly a decade ago, two small, randomized trials including both adults and children evaluated mepolizumab in the setting of EoE. Both studies demonstrated significant reductions in esophageal eosinophil counts but failed to achieve histologic remission [74,75]. Clinical results remain inconsistent, with the initial pediatric trial showing no meaningful symptom relief, while the adult study recorded only a modest yet statistically significant reduction in dysphagia, but overall results were inconsistent [74,75]. More recently, a multicentre RCT (NCT03656380) included 66 adolescents and adults with active EoE to 300 mg SC monthly for 3 months vs. placebo [76]. The primary endpoint, change in EEsAI score, was not met (−15.4 ± 18.1 vs. −8.3 ± 18.0; *p* = 0.14). Despite this, mepolizumab produced a strong biologic effect, with peak eosinophils falling from 113 to 36 eos/hpf, giving higher histologic responses (<15 eos/hpf 42%; ≤6 eos/hpf 34%) versus 3% on placebo. Extending treatment to a 6-month period maintained eosinophil suppression, but symptom scores showed no significant differences (−18.3 vs. −18.6; *p* = 0.85), and endoscopic gains were modest with no impact on fibrosis [76]. The safety profile was favorable, marked by mild injection-site reactions and no serious adverse events attributable to the drug [76]. Given the inconclusive efficacy, mepolizumab development in EoE did not progress beyond Phase 2. Thus, Mepolizumab is approved for other eosinophilic disorders like asthma and eosinophilic granulomatosis, but not for EoE.
- **Reslizumab (anti-IL-5):** Reslizumab was tested in a large Phase 2/3 trial in 226 children and adolescents with EoE [77]. Similar to other anti-IL-5, the treatment caused a significant drop in eosinophil counts (median reduction range 40–60% from baseline at highest doses) compared to minimal change on placebo. However, the therapy did not lead to full histologic remission in most patients, as no dose group achieved a median <5 eos/hpf [77]. Importantly, symptoms did not improve any more than placebo, and there were no significant differences in physician global assessment or patient symptom scores between reslizumab and placebo arms. Both coprimary endpoints, defined as eosinophil count and symptom score, were not met. In summary, while reslizumab significantly reduced esophageal eosinophilia, it failed to improve EoE symptoms [77]. After these results, reslizumab was not pursued further for EoE. Long-term follow-up showed no major safety issues over 9 years with reslizumab in patients with EoE, but also no new efficacy signal [78].
- **Benralizumab (anti-IL-5Rα):** Benralizumab is an IL-5 receptor–α antibody that not only blocks IL-5 signaling but also causes antibody-dependent cell-mediated cytotoxicity, aiding in achieving a near-complete eosinophil depletion [79]. The phase-3 MESSINA trial tested benralizumab in 211 patients aged 12–65 years with symptomatic, histologically active EoE who were randomized 1:1 to receive subcutaneous benralizumab 30 mg or placebo every four weeks for 24 weeks [80]. Histologic response, defined as ≤6 eos/hpf, was achieved in 87.4% of benralizumab recipients versus 6.5% with placebo (difference 80.8%; 95% CI 72.9–88.8; *p* < 0.001). Conversely, dysphagia improved minimally with a least-squares mean change in Dysphagia Symptom Questionnaire score that differed by only 3.0 points in favour of benralizumab (*p* = 0.18), and endoscopic and quality-of-life measures showed no meaningful advantages. Adverse events occurred in 64.1% of the benralizumab group and 61.7% of controls, with similar types and no treatment-related discontinuations [80]. The original design of this study planned that participants could enter a 28-week open-label phase with a long-term extension, yet the sponsor terminated this part of the study after week 52 because the sustained eosinophil depletion still did not yield clinically meaningful symptom relief. Among the 210 patients who had received at least one dose, 205 (98%) rolled over into the open-label period, 161 (76%) reached the week-52 visit, and 93 proceeded to the optional extension. However, 95% of these withdrew when the trial was halted, and full results from this extension remain unpublished in the peer-reviewed literature [81].

In summary, IL-5 pathway biologics have not translated into clinical success in EoE. This class of drugs consistently reduces esophageal eosinophil counts, confirming IL-5’s role in eosinophil trafficking, but has not provided meaningful symptom relief for patients [53].

#### 2.3.2. IL-13 and IL-4 Pathway Inhibitors

Interleukin-13 and IL-4 are central drivers of the allergic inflammation in EoE. IL-13, in particular, is overexpressed in EoE and induces the epithelial chemokine eotaxin-3 (CCL26), which recruits eosinophils to the esophagus. IL-4 and IL-13 together promote Th2 cell differentiation and IgE class switching. Blocking these cytokines can broadly attenuate the type 2 inflammatory response. Two approaches have been investigated: antibodies against IL-13 itself, and an antibody against the IL-4 receptor alpha (IL-4Rα), which inhibits signaling of both IL-4 and IL-13.

- **Dupilumab (anti-IL-4Rα):** Dupilumab is a fully human monoclonal antibody that binds IL-4Rα, preventing both IL-4 and IL-13 from activating their receptor pathway [82]. It is a well-established treatment for atopic dermatitis, asthma, and sinus polyposis—conditions that frequently coexist with EoE [83,84,85]. In a double-blind, randomized phase-2 trial enrolling 47 adults with active EoE, weekly subcutaneous dupilumab 300 mg for 12 weeks produced multidimensional benefits over placebo [86]. Dysphagia improved significantly (mean SDI change −3.0 vs. −1.3; ≥3-point response 39% vs. 13%). Peak oesophageal eosinophil counts fell by 96 eos/hpf (−93%) compared with a 10 eos/hpf rise (+12%) on placebo. Endoscopic activity regressed (EREFS −1.9 vs. −0.3) and oesophageal distensibility increased 18% while remaining static in controls. Adverse events were mainly mild injection-site erythema (35%) and nasopharyngitis (22%), with no serious drug-related events or deaths [86]. Building on this, the Phase 3 program (LIBERTY-EoE trials) was conducted in patients aged ≥12. In two independent 24-week placebo-controlled trials (Part A and Part B, *n* ≈ 80 each), dupilumab 300 mg weekly produced high rates of histologic remission and clinical improvement. In Part A, 60% of dupilumab-treated patients achieved histologic remission (≤6 eos/hpf) compared to only 5% on placebo. Similarly, 59% vs. 6% achieved remission in Part B [87]. Dupilumab also significantly improved symptoms: patients experienced a 21–22 point improvement (reduction) in their Dysphagia Symptom Questionnaire (DSQ) scores, versus a 10-point improvement on placebo [88]. The dupilumab–placebo difference of 12 points was clinically meaningful, reflecting substantial symptom relief by 24 weeks [88]. These positive results were published and led to USFDA approval in May 2022 as the first treatment for EoE [44]. Dupilumab is approved in the US, EU, and other regions for adolescents and adults with EoE, and in 2023, the USFDA expanded its indication to include children as young as 1 year (based on pediatric trial data). In clinical practice, dupilumab has become a breakthrough option—inducing histologic remission in 60% of patients and significantly improving dysphagia, while being generally well tolerated. Common side effects include injection site reactions and peripheral blood eosinophilia (transient), and in other conditions, dupilumab is associated with rare conjunctivitis or arthritic reactions, but overall, it has a favorable safety profile with no immunosuppression [87,88]. The success of dupilumab confirms that blocking IL-4/IL-13 can effectively treat EoE [82].
- **Cendakimab (Anti-IL-13):** Cendakimab (formerly called RPC4046, also code-named CC-93538) is a humanized monoclonal antibody that antagonizes IL-13 by preventing it from binding to both IL-13 receptor α-1 and α-2 subunits [89]. In a Phase 2 trial of adults with active EoE (RPC4046-EoE Study), cendakimab showed strong biologic activity: after 16 weeks of weekly therapy, patients had a reduction of nearly 100 eos/hpf in peak esophageal eosinophil counts from baseline, whereas placebo had essentially no change. Endoscopic improvement was also notable—the treatment group had significant regression of features of the EREFS score, compared to placebo. On symptoms, the Phase 2 showed a positive trend: dysphagia symptom scores improved more in the 360 mg cendakimab group than placebo, but the difference fell just short of statistical significance at 16 weeks (*p* = 0.073) [89]. Nonetheless, given the marked histologic and endoscopic efficacy, the trial was considered positive. Cendakimab was well tolerated in Phase 2, with headache and upper respiratory infections the most common adverse events with a similar rate to placebo [89]. In an open-label extension trial, long-term treatment with cendakimab was generally well tolerated and led to sustained or improved endoscopic, histologic, and clinical outcomes over 52 weeks, supporting its potential as a maintenance therapy for eosinophilic esophagitis. The Phase 3 trial of cendakimab in EoE (a large multinational study enrolling 430 patients aged 12–75) was recently completed, and results are available on clinicaltrials [90]. In this trial, patients received cendakimab 360 mg either every week or every other week (after an initial 24-week induction) vs. placebo. Both weekly and every-other-week cendakimab regimens significantly reduced dysphagia frequency (mean reduction of 4.7 days vs. 2.7 days with placebo) and achieved histologic remission (≤6 eos/hpf) in 36–39% of patients compared to 0.9% with placebo at week 24. These effects were maintained through week 48, with continued symptom improvement and sustained histologic response. The safety profile was favorable, with no new safety signals and a low incidence of serious adverse events [90].
- **Dectrekumab (Anti-IL-13):** Dectrekumab (QAX576) is a fully human monoclonal antibody targeting IL-13. In a proof-of-concept Phase 2 trial (adult EoE patients), intravenous dectrekumab achieved significant histological improvements: mean esophageal eosinophil counts dropped 60% from baseline (versus a 23% increase with placebo, *p* = 0.004) [91]. The drug’s administration showed a trend toward improved dysphagia symptoms compared with placebo. However, the predefined primary endpoint (≥75% eosinophil reduction in a set proportion of patients) was not met, and complete histologic remission was not achieved. Dectrekumab was generally well tolerated, with no serious adverse events reported [91].

The success of dupilumab marks a paradigm shift in EoE therapy, offering a valid option that induces both histological and symptomatic remission. These agents target upstream cytokines, thereby silencing multiple inflammatory pathways: not only eosinophils, but also affecting mast cells, basophils, IgE, and fibroblasts involved in EoE pathology [92]. Ongoing research will clarify the optimal positioning of these biologics, clarifying how to use them early in high-risk cases to prevent fibrosis.

#### 2.3.3. Thymic Stromal Lymphopoietin

Upon allergen exposure, esophageal epithelial cells release thymic stromal lymphopoietin (TSLP), an alarmin that conditions dendritic cells and activates type-2 innate lymphoid cells. Antigen-charged dendritic cells steer naïve CD4^+^ T cells toward Th2 differentiation, while ILC2s immediately secrete IL-5 and IL-13. A positive loop amplifies IL-4, IL-5, and IL-13, driving eosinophil influx, barrier dysfunction, and remodeling. Blocking TSLP disrupts this cascade, a principle validated by anti-TSLP therapy in asthma clinical trials [93].

- **Tezepelumab:** Tezepelumab has demonstrated clinical efficacy in severe asthma, earning USFDA approval for this indication. Its elevated presence in EoE biopsy samples suggests it may also help sustain the local Th2-skewed inflammatory milieu. The CROSSING trial (NCT05583227) is an ongoing, randomised, double-blind, placebo-controlled, multicentre trial in 360 adolescents and adults aged 12–80 years with symptomatic, histologically active EoE [94]. Participants receive subcutaneous tezepelumab or placebo every four weeks for a 52-week double-blind period. Co-primary end-points are histologic remission (≤6 eosinophils per high-power field) and change in Dysphagia Symptom Questionnaire (DSQ) score at week 16; key secondary outcomes include endoscopic improvement, quality-of-life scores, steroid-free remission and maintenance of response through week 52. An optional open-label extension will follow to collect long-term safety and durability data through January 2027, the planned primary-completion date. The trial is recruiting across North America, Europe and Asia-Pacific [94]. No results have yet been posted, but a 2024 case report described complete clinical and transcriptomic remission in a teenager with steroid-refractory EoE who received tezepelumab for co-existing asthma [95].
- **Solrikitug:** Solrikitug is a next-generation human IgG1 monoclonal antibody against TSLP. Its efficacy in EoE is being tested in ALAMERE (NCT06598462), a phase 2, randomised, double-blind, placebo-controlled study that began on 16 October 2024 and is currently recruiting around 157 adults with symptomatic, histologically active EoE at about 80 centres across North America, Europe, Asia-Pacific and Australia [96]. Participants receive one of three subcutaneous dose levels of solrikitug or a matching placebo every four weeks for a 24-week blinded treatment period; completers may roll into a 28-week open-label extension, after which a 16-week safety follow-up brings total participation to 68 weeks. Co-primary endpoints at week 24 are histologic remission defined as peak oesophageal eosinophil density ≤ eos/hpf and change from baseline in the 14-day DSQ, while ranked secondary outcomes examine endoscopic and detailed histologic scores, steroid-free clinico-histologic remission, durability of response through week 52, and standard safety, pharmacokinetic and immunogenicity parameters. Top-line primary data are expected by 30 April 2027, with final database lock targeted for 31 August 2027, and no results have yet been reported [96].

#### 2.3.4. Sialic-Acid-Binding Immunoglobulin-like Lectin (Siglec)-8

Sialic-acid-binding immunoglobulin-like lectin-8 is an inhibitory receptor present almost solely on human eosinophils and mast cells. When glycans or antibodies cluster Siglec-8, two tyrosine sites inside the receptor gain phosphate groups and attract the phosphatases SHP-1 and SHP-2. In eosinophils, this starts a burst of reactive oxygen, switches on caspases and ends in apoptosis, while in mast cells, it turns off the kinases Lyn and Syk, stops calcium entry and blocks degranulation driven by immunoglobulin E. These mechanisms lead to the rapid elimination of eosinophils and the functional silencing of mast cells, thereby offering a highly selective means to dampen allergic and eosinophil-driven inflammation without broadly suppressing the immune system [97,98].

- **Lirentelimab (AK002):** Lirentelimab is a humanised IgG1 monoclonal antibody that binds Siglec-8. The KRYPTOS trial (NCT04322708) is a multicentre, randomised, double-blind, placebo-controlled phase 2/3 study in 277 adolescents and adults aged 12–80 years with symptomatic, histologically active EoE. Participants received six monthly intravenous infusions of high-dose lirentelimab (1 mg kg^−1^ loading, then 3 mg kg^−1^), low-dose lirentelimab (1 mg kg^−1^ throughout) or placebo over a 24-week double-blind period, with an optional 24-week open-label extension for long-term follow-up [99]. Co-primary end-points were histologic remission (≤6 eos/hpf) at week 24 and change in mean daily Dysphagia Symptom Questionnaire (DSQ) score over weeks 23–24; secondary outcomes included endoscopic improvement, quality-of-life measures and safety [99]. The study met its histologic target, with remission in 88% (high dose) and 92% (low dose) versus 11% with placebo, but the symptom co-primary end-point was not reached (least-squares mean DSQ change −17.4, −11.9 and −14.6, respectively). Adolescents showed a numerically greater DSQ improvement (−18.4 and −16.4 vs. −8.9). Infusion-related reactions and headache were the most frequent adverse events; only three serious adverse events occurred across all arms [100]. Although an open-label extension is continuing to gather durability and safety data, the discordance between robust tissue responses and modest symptomatic benefit has tempered enthusiasm for this approach.

#### 2.3.5. Immunoglobulin E (IgE) Neutralization

IgE orchestrates immediate hypersensitivity: IL-4/IL-13–driven class switching yields IgE that occupies high-affinity FcεRI on mast cells and basophils. Allergen-induced cross-linking triggers rapid degranulation, histamine release and cytokine production, recruiting eosinophils and sustaining Th2-dominated inflammation in atopic disorders such as asthma.

- **Omalizumab:** Omalizumab is a humanised IgG1 monoclonal against IgE-driven inflammation. Two clinical studies have explored its role in eosinophilic oesophagitis (EoE). A 12-week, open-label, single-centre phase 1b pilot study enrolled 15 adolescents and adults (median age 14 years, range 12–71) who received weight- and IgE-adjusted subcutaneous omalizumab every 2–4 weeks [101]. Histological-clinical remission (≤15 eos) was achieved in 33% of patients, with significant falls in peak eosinophil counts from 31 to 19 eos/hpf (*p* = 0.034), mast-cell density and symptom scores, and no serious adverse events. A 16-week, randomised, double-blind, placebo-controlled phase 2 trial allocated 30 mostly adult patients to omalizumab (*n* = 16) or placebo (*n* = 14) every 2–4 weeks [102]. Omalizumab produced no significant reduction in eosinophil burden or dysphagia scores, despite effective depletion of tissue IgE, indicating that IgE is not a dominant driver of EoE in this population. Collectively, these findings imply that while anti-IgE therapy can induce remission in a paediatric-leaning subgroup with low peripheral eosinophilia, its overall efficacy in adult EoE appears limited.

#### 2.3.6. Mycobacterium Tuberculosis Chaperonin 60.1 Peptide

Mycobacterium tuberculosis chaperonin 60.1–derived peptide 104 binds CD14/TLR2 on monocytes, reprogramming them toward regulatory phenotypes that suppress Th2 cytokines, eotaxin, and IL-13; this limits eosinophil recruitment, reduces epithelial barrier damage, and restores immune homeostasis within the inflamed esophagus, promoting mucosal tolerance [103].

- **IRL201104:** also known as “1104” is a first-in-class synthetic peptide derived from Mycobacterium tuberculosis chaperonin 60.1. A Phase 2a trial with a 2-week regimen of three doses in active EoE demonstrated promising results [103]. IRL201104 led to a 50% reduction in peak esophageal eosinophil counts from baseline and significantly improved patient-reported dysphagia symptom scores compared to placebo, with relief persisting 4 weeks beyond the last dose [103]. Treated patients showed broad immunologic shifts, including normalization of key EoE-related cytokine transcripts and markers of barrier function. The peptide was well tolerated, with no serious adverse events or any treatment discontinuations due to drug-related effects [103]. On the strength of Phase 2a data, IRL201104 received USFDA Orphan Drug Designation for EoE. A longer Phase 2b trial, using higher doses and extended dosing, is planned to further assess efficacy and durability in 2025 [104].

#### 2.3.7. IL-15 Pathway Inhibitors

In eosinophilic esophagitis, interleukin-15 released by stressed epithelial and immune cells fuels a self-amplifying inflammatory niche (PMCID: PMC5540113): it licenses dendritic cells, extends survival of cytotoxic T and NK cells, and primes eosinophils alongside ILC2s, thereby boosting type-1 and type-2 cytokine output while crippling regulatory brakes. CALY-002, a neutralizing anti-IL-15 antibody, is designed to sever these converging signalling hubs, curb eosinophil recruitment and activation, and shield the esophageal mucosa from fibrotic remodeling.

- **CALY-002** is a humanized monoclonal antibody against interleukin-15 (IL-15), a cytokine that drives T-cell and innate lymphocyte activation. A Phase 1a/1b trial tested single doses in healthy volunteers and multiple doses in EoE patients [105]. In healthy subjects, CALY-002 was well tolerated and demonstrated clear on-target activity (dose-dependent reductions in NK cell counts, reflecting IL-15 blockade) [105]. Preliminary results in EoE patients showed promising efficacy. CALY-002 markedly reduced esophageal eosinophil density and histologic damage while improving dysphagia symptoms in steroid-refractory EoE. No serious adverse events were observed; early studies report a good overall safety profile for IL-15 inhibition in EoE. CALY-002 holds orphan designation for EoE in both the EU and the US.

#### 2.3.8. Sphingosine-1-Phosphate Receptor (S1P) Modulator

Etrasimod is an oral, next-generation modulator with high selectivity for S1P 1, 4 and 5. By transiently trapping T-lymphocytes in lymph nodes, it lowers their trafficking to the esophageal mucosa, dampens type-2 cytokine and eotaxin signals that drive eosinophil recruitment, limits epithelial barrier injury, and thereby helps re-establish immune homeostasis and durable mucosal healing in EoE [20,106].

- **Etrasimod (APD334):** This drug is a once-daily oral S1P 1/4/5 modulator and in the phase 2b VOYAGE study (NCT04682639) involving 108 adults with active EoE, the 2 mg dose for 24 weeks produced a 52.4% reduction in peak esophageal eosinophil counts versus a 61% increase on placebo at week 24, after meeting the primary endpoint at week 16 (−46% versus baseline) [107]. Histologic remission (<15 eos/hpf) was achieved in ~33% of patients and a significant improvement in EREFS (−1.3 points) and patient-reported outcomes, including a −21.6-point fall in the DSQ among participants without prior dilation. The therapy showed a favorable safety profile, with mainly mild or moderate events (transient first-dose bradycardia, modest transaminase rises) and no study withdrawals for drug reasons. Benefits in histology, endoscopy and symptoms were maintained through week 52 in the open-label extension, with no new safety signals [107]. On 8 June 2021, the molecule received USFDA Orphan Drug Designation for EoE [108], but the manufacturer has halted development for this indication. In the 2025 pipeline update dated 29 April 2025, etrasimod for EoE is listed among the programs discontinued from development, and no Phase 3 trial is registered [109].

#### 2.3.9. Anti-KIT Monoclonal Antibody

Barzolvolimab is a humanized monoclonal antibody that binds the KIT receptor on mast cells, inhibiting this tyrosine kinase required for mast cell survival and activation [110]. Targeted mast-cell depletion with barzolvolimab—directed at cells now recognized as pivotal drivers of eosinophilic esophagitis—is intended to dampen downstream type 2 inflammation, curb eosinophil recruitment and epithelial injury, and ultimately restore immune homeostasis within the esophageal mucosa [111].

- **Barzolvolimab (CDX-0159):** This is a first-in-class mast cell–depleting antibody under investigation for EoE. A Phase 2 trial (“EvolvE”, NCT05774184) in patients with active EoE is evaluating subcutaneous barzolvolimab 300 mg every 8 weeks versus placebo in a 16-week double-blind period, followed by a 12-week open-label extension [112]. The primary endpoint is a reduction in peak esophageal intraepithelial mast cell density at 12 weeks, with key secondary endpoints including improvements in dysphagia symptom scores and peak eosinophil counts [112]. Screening biopsies from 117 candidates showed a strong correlation between mast-cell and eosinophil densities, supporting KIT blockade as a mechanistic strategy in EoE [113]. Enrollment concluded in February 2025, and topline efficacy data are expected in the second half of 2025 [112]. If the Phase 2 outcomes are positive, this therapy could represent a novel treatment avenue in EoE by addressing a previously under-recognized driver of disease.

#### 2.3.10. α1-Proteinase Inhibitor

In eosinophilic esophagitis, loss of the epithelial antiprotease SPINK7 unleashes kallikrein-5, which cleaves PAR2 and sparks a protease-activated danger circuit [114]: the KLK5–PAR2 axis erodes barrier integrity, triggers alarmin release, and amplifies eosinophil-recruiting type-2 cytokines while bypassing conventional regulatory checkpoints. Zemaira—plasma-purified α1-antitrypsin (A1PI)—may help restore the antiprotease shield, quench KLK5 activity, and re-establish mucosal homeostasis before fibrosis can take hold [114].

- **Zemaira** (α1-proteinase inhibitor, human) is an intravenous, plasma-derived A1PI long used for α1-antitrypsin deficiency. The Phase 2 open-label Zemaira Eosinophilic Esophagitis Pilot Study (ZEEPS, NCT05485155) is now recruiting adults with active, treatment-refractory EoE at NIH and Cincinnati Children’s. Participants receive 120 mg kg^−1^ IV weekly for 8 weeks (follow-up 12 weeks); primary read-outs include peak eosinophil density and dysphagia scores. Total planned enrollment is 15 [115]. Decades of augmentation-therapy experience and pre-clinical data showing A1PI reversal of KLK5-driven pathology support a favorable safety outlook and a protease-targeted, steroid-sparing strategy for EoE [114].

## 3. Challenges in Eosinophilic Esophagitis Clinical Trials and Potential Solutions

Since its recognition as a distinct entity in the early 1990s, our understanding of EoE has evolved significantly. However, this evolution has been accompanied by increasing complexity in designing and implementing effective clinical trials. EoE clinical trial design faces multifaceted challenges that need to be addressed in order to advance the field toward more meaningful therapeutic development.

### 3.1. Symptom-Histology Discordance: A Fundamental Challenge

Perhaps the most important challenge in EoE clinical trials is the frequent discordance between patient-reported symptoms and histologic findings [116,117]. Trials of potent eosinophil-depleting agents have demonstrated this striking disconnection. The KRYPTOS trial evaluated lirentelimab, an antibody against siglec-8 that depletes eosinophils and inactivates mast cells [100]. Despite achieving extraordinarily high rates of histologic response (≤6 eosinophils per high-power field [HPF] in 88–93% of treated patients compared to 11% with placebo), the medication failed to improve dysphagia symptoms compared to placebo. Similarly, in the MESSINA trial, benralizumab, an antibody against the IL-5 receptor, demonstrated impressive histologic efficacy (87% histologic response vs. 7% with placebo) but no significant improvement in symptoms or endoscopic appearance [80].

While the eosinophil has been the defining cell type and histologic marker of the disease, it may not be the primary driver of symptoms or tissue damage [118]. Instead, it appears to be one component of a complex inflammatory cascade involving multiple cell types and mediators [119]. The allergic or environmental triggers, persistent barrier dysfunction, activated T cells and their associated cytokine cascade, and other inflammatory cells, including mast cells, fibroblasts, and basophils, likely continue to drive disease activity even when eosinophils are eliminated from the tissue.

Recent research has quantified this phenomenon. A retrospective cohort study by Beveridge et al. found that nearly half (46%) of EoE patients in histologic remission continued to experience esophageal symptoms, with dysphagia being the most common persistent complaint (41.2% of patients) [120]. This discordance appears to be influenced by multiple factors, such as fibrostenotic disease and psychological factors. In fact, the same study found that anxiety was associated with a nearly four-fold increased risk of persistent dysphagia (adjusted odds ratio 3.77) even after controlling for other factors.

This discrepancy fundamentally complicates trial design, endpoint selection, and interpretation of results, as well as clinical practice. Using eosinophil counts alone as the primary outcome measure for EoE trials is clearly insufficient. Regulatory authorities themselves diverge in their emphasis. The USFDA has typically required co-primary endpoints in registration trials, including both histologic improvement (≤6 eosinophils per HPF) and symptomatic improvement (typically measured by the Dysphagia Symptom Questionnaire [DSQ]) [121]. The European Medicines Agency (EMA), in contrast, has placed greater emphasis on mucosal healing and overall symptom relief without specific numeric thresholds for eosinophil counts. This regulatory divergence creates challenges for global drug development programs.

Furthermore, the field would benefit from patient stratification strategies that account for disease phenotype. As highlighted in a recent review by Ruffner and Cianferoni, EoE, like other atopic disorders, demonstrates diverse clinical presentations (phenotypes) based on response to therapy, natural history, and association with atopic comorbidities [122]. These varying phenotypes likely reflect different pathogenetic mechanisms (endotypes), including T Helper type 2 inflammation, epithelial barrier defects, enhanced fibrosis, and association with rare monogenetic diseases. The recognition of these distinct phenotypes and endotypes is crucial for advancing precision medicine approaches in EoE. A further complexity was identified by Shoda et al., who used machine learning to examine histological, endoscopic, and molecular features in EoE patients [123]. They identified three distinct endotypes: EoE-1, representing 35% of patients with mild features and steroid-responsiveness; EoE-2, representing 29% of patients with pediatric-onset, steroid-refractory disease with high expression of inflammatory genes; and EoE-3, representing 36% of patients with low expression of epithelial differentiation genes and greater frequency of narrow-caliber esophagus. These endotypes may account for diverse natural history and treatment responses, complicating trial design and interpretation. Similarly, disease duration, prior treatment history, and the presence of psychiatric comorbidities should be considered in trial design and analysis.

### 3.2. Endpoint Selection and Validation

The selection of appropriate endpoints remains one of the most challenging aspects of EoE trial design. A comprehensive approach to endpoint selection should consider multiple domains. While eosinophil counts remain important, broader assessment of histologic features (including basal zone hyperplasia, spongiosis, and lamina propria fibrosis) may provide more holistic evaluation of tissue response [92]. Standardized endoscopic assessment using validated tools such as the EoE Endoscopic Reference Score (EREFS) can capture both inflammatory and fibrostenotic features [124]. The ASCENT (Assessment of Clinical endpoints in Eosinophilic esophagitis for Novel Therapeutics) consensus, convened specifically following recent trial failures where eosinophil-depleting biologics achieved near-complete tissue eosinophil elimination without clinical benefit, proposes a fundamental paradigm shift from peak eosinophil count toward comprehensive assessment using the EoE Histology Scoring System (EoE-HSS) and EREFS score, with emphasis on “clinicopathologic” response where both symptom-based and biological outcomes must improve in the same patient [92]. Objective measures of esophageal function, such as distensibility assessment using the functional lumen imaging probe (FLIP), offer insights into the mechanical consequences of inflammation and fibrosis that may more directly relate to symptoms [125,126]. Recent research by Carlson et al. has demonstrated that objective physiomechanical assessments using FLIP can identify distinct EoE phenotypes with clinical relevance [127]. Their PhysioMechanical classification model incorporates measurements of esophageal distensibility, compliance, contractile response, and EGJ function to categorize patients into seven distinct phenotypes ranging from normal to nonreactive fibrostenosis. These classifications correlate with disease duration, endoscopic features, and treatment response, offering potential prognostic value for clinical trials.

Similarly, Carlson et al. developed the C2D2 score, a composite measure that quantifies physiomechanical esophageal dysfunction in EoE [126]. This score significantly correlates with disease markers, including mucosal eosinophil count and EREFS score and has demonstrated predictive value for PPI response that outperforms traditional measures. Such objective assessments of esophageal remodeling may provide valuable outcome measures beyond histology.

Validated patient-reported outcome instruments such as the Dysphagia Symptom Questionnaire (DSQ) or EoE Activity Index (EEsAI) capture the symptomatic burden experienced by patients. The Esophageal Hypervigilance and Anxiety Scale (EHAS) can assess the psychological aspects of the disease that may influence symptom perception. Assessment of adaptive behaviors at mealtime, such as those measured by the Pisa EoE Adaptation Questionnaire, may also be valuable in spotting hidden signs of disease activity. Recent research by Visaggi et al. highlights the importance of accounting for adaptive behaviors when assessing treatment outcomes [128]. Their study demonstrated that approximately 77% of patients with histologically active EoE exhibited adaptive eating behaviors (compared to 26% in remission), and these behaviors significantly modified symptom reporting. Similarly, de Rooij et al. found that adaptive behaviors were prevalent in EoE populations and negatively impacted quality of life, particularly in the domains of “eating/diet impact” and “disease anxiety.” [129,130]. These findings suggest that measurements of adaptive behaviors provide complementary clinical information not detected by symptoms alone and should be considered as secondary endpoints in clinical trials.

Given the multifaceted nature of EoE, composite endpoints that incorporate elements from multiple domains may provide the most comprehensive assessment of treatment effects. Moving beyond traditional fixed-timepoint assessments, the recent ASCENT consensus advocated for time-to-event designs that capture clinically meaningful disease progression [92]. Rather than measuring eosinophil counts at week 24, these approaches would follow patients until they experience significant clinical events such as first food impaction, need for esophageal dilation, disease relapse after treatment discontinuation, or stricture development. As we advance therapeutic development for EoE, continued dialogue among researchers, pharmaceutical companies, regulatory agencies, and patient advocates remains essential to redefine success criteria that truly benefit patients living with this challenging condition.

### 3.3. Recruitment and Inclusion Challenges: Expanding the Trial Population

Clinical trials in EoE face substantial challenges related to patient recruitment and inclusion criteria, which can limit both trial feasibility and the generalizability of results. Additionally, the chronic nature of EoE often necessitates long-term trials, increasing the risk of dropout and loss to follow-up. Moreover, the global landscape of EoE research reveals significant disparities in recognition, understanding, and research infrastructure [131]. While EoE was initially described predominantly in North American and European populations, reports from Asia, the Middle East, Latin America, and Africa demonstrate its global presence, albeit with varying prevalence.

Strategies to enhance recruitment and retention include consortium-based approaches that leverage networks of specialized centers to facilitate enrollment by accessing larger patient populations. Examples include the Consortium of Eosinophilic Gastrointestinal Disease Researchers (CEGIR) in the United States and the European Society of Eosinophilic Oesophagitis (EUREOS) in Europe. Involvement of patient advocacy organizations such as the American Partnership for Eosinophilic Disorders (APFED) and the Campaign Urging Research for Eosinophilic Disease (CURED) can enhance awareness and motivation for trial participation.

These challenges have multiple dimensions, ranging from regulatory requirements to the complexities of the disease itself.

Regulatory agencies often require stringent inclusion criteria, including high symptom and histologic thresholds, to ensure that trial populations truly have active disease. While scientifically sound, this approach leads to high screening failure rates and potentially excludes patients who might benefit from therapy. The disconnect between clinical and endoscopic disease activity further complicates patient selection. A patient with significant endoscopic disease but minimal symptoms may be excluded from trials despite having objectively severe disease that warrants treatment. Inclusion of patients with varying disease characteristics may increase generalizability but can dilute treatment effects. Moreover, patients with severe fibrostenotic disease, frequent dilation requirements, or inability to discontinue existing therapies may be excluded, such as PPI-naïve, patients typically cannot undergo trials without first demonstrating PPI failure. Conversely, highly selective criteria may enhance the likelihood of demonstrating efficacy but limit applicability to real-world populations.

As more pharmaceutical companies develop EoE treatments, competition for eligible patients intensifies. The average recruitment rate for clinical trials may decline significantly over time, slowing trial completion and potentially increasing costs. To address these challenges, the field needs to develop more pragmatic trial designs that better reflect real-world patient populations while maintaining scientific rigor. This includes less restrictive inclusion criteria, stratification based on psychiatric comorbidities, and inclusion of patients with varying disease durations and phenotypes.

Patient-centered recruitment strategies are also essential. The PassITON trial for COVID-19 demonstrated that multicultural and multilingual awareness-raising strategies, community engagement, and flexible trial design can significantly improve recruitment among underrepresented populations [132]. Similar approaches could enhance EoE trial enrolment and diversity. Decentralizing certain trial components through remote symptom monitoring, telemedicine visits where appropriate, and mobile research teams could further expand access, particularly for patients in remote areas or with limited healthcare access [133,134].

Standardization of endpoint assessment through centralized reading of endoscopic images and histopathologic specimens can improve consistency across trial sites and regions, enhancing both scientific validity and operational efficiency [135]. This approach is particularly valuable as trials expand globally to include sites with varying levels of experience in EoE assessment and management.

### 3.4. Ethical Considerations in Placebo Use: Balancing Science and Patient Care

The use of placebo controls in EoE trials presents increasingly complex ethical challenges as effective treatments become available [136]. Historically, placebo-controlled trials have been the gold standard for demonstrating efficacy, and regulatory agencies often continue to require this design for approval of new medications [137]. There are valid scientific reasons for this requirement, including the fluctuating nature of EoE symptoms, the potential for spontaneous improvement, the substantial placebo effect observed in many gastrointestinal conditions, and the need for a clear demonstration of benefit [138].

However, as the therapeutic landscape evolves, withholding effective treatment from patients with a chronic, potentially progressive disease raises significant ethical concerns [139]. Patients assigned to a placebo may be exposed to unnecessary risks, including disease progression, food impaction, and reduced quality of life [140]. These concerns are particularly acute for patients with severe symptoms or those who have failed multiple prior therapies.

From a practical perspective, patient preferences are also shifting. As patients gain access to effective treatments in clinical practice, their willingness to participate in placebo-controlled trials diminishes. This changing landscape necessitates innovation in trial design to balance scientific rigor with ethical considerations and practical feasibility.

Several alternative approaches merit consideration. Adaptive trial designs can minimize placebo exposure by allowing for early termination of ineffective treatments or transition to active therapy based on interim analyses [141]. Bayesian statistical approaches incorporate prior knowledge to reduce required sample sizes and potentially decrease the number of patients receiving placebo [142]. Multi-arm, multi-stage trials or seamless Phase 2/3 designs can evaluate multiple treatments or doses simultaneously, increasing the proportion of patients receiving active therapy [143].

Active comparator trials, which compare new treatments directly to established therapies rather than a placebo, are increasingly relevant as the field matures [144]. These designs can address clinically relevant questions about relative efficacy and safety while ensuring all participants receive active treatment. Add-on designs, where a new treatment is added to standard care and compared to standard care alone, may be particularly appropriate for patients with refractory disease [145].

Crossover designs, in which patients receive both active treatment and placebo for defined periods, allow all participants to access active therapy while maintaining some elements of placebo control [146]. This approach may be particularly useful for evaluating symptom-based outcomes. Additionally, predefined early escape criteria can allow patients not responding to placebo to receive active treatment, reducing the ethical concerns of prolonged placebo exposure [92].

As the field continues to evolve, a thoughtful balance between scientific rigor, regulatory requirements, ethical considerations, and patient preferences will be essential in designing EoE clinical trials that advance therapeutic development while respecting patient needs.

### 3.5. Psychiatric Comorbidities: The Overlooked Dimension

The psychological dimension of EoE has emerged as a critical factor in disease experience and treatment outcomes, yet it remains largely unaddressed in clinical trial design and analysis. Research has consistently demonstrated high rates of anxiety, depression, and hypervigilance among EoE patients, with profound implications for symptom perception, reporting, and response to therapy.

Anxiety is associated with an increased risk of persistent dysphagia in patients with histologic remission [147]. Even more strikingly, in patients without endoscopic fibrostenosis, anxiety was the only significant predictor of persistent dysphagia [120]. Similarly, anxiety and depression were the primary predictors of persistent heartburn and chest pain, with extraordinary odds ratios exceeding 10 in multivariate analyses. These findings suggest that psychological factors may be equally or more important than traditional disease measures in determining symptomatic outcomes.

The relationship between psychological factors and EoE also highlights the need for multidisciplinary approaches to both research and clinical care. Collaboration between gastroenterologists, allergists, psychiatrists, and psychologists could yield a more comprehensive understanding and management strategies.

Future clinical trials should incorporate standardized assessment of psychiatric comorbidities and stratification or post hoc analyses based on psychiatric status could identify differential treatment effects and inform personalized therapeutic approaches.

### 3.6. Diet Therapy Trials: Unique Challenges and Opportunities

Dietary elimination represents one of the most effective approaches for treating EoE, yet clinical trials of dietary interventions face distinct challenges compared to pharmacologic studies [148]. These challenges span scientific, practical, and psychological dimensions, creating barriers to rigorous evaluation and clinical implementation.

From a scientific perspective, dietary trials are difficult to blind effectively. This limitation introduces potential bias in both patient-reported outcomes and investigator assessments.

Moreover, adherence to restrictive diets is notoriously difficult to maintain, particularly over extended periods [149]. Unlike medication trials, where pill counts can provide objective adherence measures, dietary compliance relies heavily on self-reporting. Cross-contamination and trace exposure to eliminated allergens can occur inadvertently, potentially confounding results. Moreover, the financial and logistical burdens of specialized diets can create barriers to participation and adherence, particularly for socioeconomically disadvantaged populations.

The psychological impact of dietary restriction introduces additional complexities. As documented in recent research, restrictive diets can significantly affect quality of life through impacts on social functioning, food-related anxiety, and the burden of food preparation [150]. More concerning, emerging evidence suggests associations between elimination diets and disordered eating patterns, including Avoidant/Restrictive Food Intake Disorder (ARFID) [151]. Despite these challenges, dietary trials offer unique opportunities to advance both patient care and scientific understanding of EoE. Recent research has demonstrated that less restrictive approaches may achieve efficacy comparable to more restrictive six-food elimination diets in many patients [31]. These findings suggest potential paths to maintain efficacy while reducing burden but require further validation in rigorous clinical trials.

Future dietary intervention studies should incorporate several innovative approaches. Novel endpoints beyond traditional histologic and symptomatic measures, such as nutritional adequacy, burden of implementation, and sustainability, would provide a more holistic assessment of dietary interventions.

The role of registered dietitians as integral members of the research team cannot be overstated [152]. Their expertise can enhance protocol development, participant education, adherence assessment, and nutritional monitoring throughout trials.

As pharmaceutical options for EoE expand, comparative effectiveness research examining dietary versus pharmacologic approaches, alone or in combination, will become increasingly important [32]. Such studies should consider not only traditional efficacy measures but also patient preferences, quality of life impact, long-term sustainability, and cost-effectiveness.

### 3.7. Long-Term Disease Modification: The Frontier of EoE Research

As EoE is recognized as a chronic, often progressive disorder, shifting research focus toward long-term disease modification represents perhaps the most ambitious frontier in clinical trials [153]. The natural history of EoE reveals a chronic, progressive disorder that, if left untreated, may evolve from an inflammatory to a fibrostenotic phenotype. Chang et al. demonstrated that patients with EoE who experienced a gap in care of two or more years had markedly increased endoscopic severity scores (2.4 vs. 1.5; *p* < 0.001) and smaller esophageal diameters (11.0 vs. 12.7 mm; *p* = 0.04) compared to pre-gap assessments [154]. Importantly, each additional year without proper care increased the odds of stricture development by 26%, even after accounting for pre-gap dilation. The work of Araujo et al. further supports the value of these measurements by demonstrating that esophageal distensibility measured by FLIP decreases with longer disease duration and diagnostic delay [125]. Their research showed that abnormal esophageal distensibility (defined as DP ≤ 17 mm) was increasingly prevalent with disease duration: from 23% in patients with <5 years of symptoms to 64% in those with ≥25 years, providing objective evidence of progressive fibrostenotic remodeling in EoE.

This complicates trial design in more ways. First, the trial population may include patients at different points along this disease spectrum, from newly diagnosed cases with primarily inflammatory features to long-standing cases with established fibrosis. Second, the timeframe required to demonstrate meaningful changes in fibrostenotic features may be considerably longer than what is practical for clinical trials. Third, outcome measures need to capture both inflammatory and fibrotic elements of the disease, as done by the EREFS.

While current therapies effectively control inflammation and symptoms when administered continuously, evidence for true disease-modifying effects, those that alter the natural course of EoE after treatment discontinuation, remains limited [22].

Long-term trials in EoE face formidable challenges [14]. The progression from inflammatory to fibrostenotic phenotypes occurs over years or decades in many patients, necessitating extended study durations that exceed typical trial timeframes. Furthermore, ethical considerations limit the feasibility of long-term placebo-controlled studies in a disease with established effective therapies.

Despite these challenges, several lines of evidence suggest that disease modification may be achievable. Early intervention appears associated with better outcomes and lower rates of fibrostenotic complications. Certain therapies, particularly those targeting upstream mediators like IL-4 and IL-13, show potential to reverse established tissue remodeling in addition to controlling inflammation [71]. Moreover, emerging data suggest that proper control of inflammation can reduce the need for repeated esophageal dilations, suggesting modification of the fibrostenotic disease trajectory [155].

A potential solution would be the use of a reliable biomarkers that specifically indicate fibrosis and stenosis progression, preferably a serum biomarker, in order to non-invasively and repeatedly assess treatment response. FLIP is moving in this direction, as well as molecular markers in esophageal biopsies and advanced imaging techniques to quantify esophageal wall thickening and fibrosis. Given this critical knowledge gap, extended follow-up of patients from existing clinical trials after treatment discontinuation could provide valuable insights into the durability of response without the ethical concerns of primary placebo assignment [156]. Prospective registries with standardized assessments could characterize long-term outcomes across different treatment approaches while accommodating real-world variability in management [157]. Notably, the phase IV REMODEL trial (NCT06101095) is prospectively evaluating whether long-term dupilumab mitigates fibrostenotic progression in adult EoE, with EndoFLIP-derived esophageal distensibility as the primary endpoint and longitudinal histologic and molecular assessments [157].

### 3.8. Pediatric-Specific Considerations

Pediatric EoE trials face additional challenges beyond those encountered in adult studies [158]. Children at different developmental stages may present with varying symptoms and have different abilities to report subjective experiences. Interventions must consider potential impacts on growth and development, particularly for dietary restrictions or medications affecting appetite. The informed consent process involves parents/guardians while also requiring age-appropriate assent from pediatric participants. Moreover, important differences between pediatric and adult EoE must be acknowledged, as therapeutic responses, pharmacokinetics, and optimal dosing regimens may vary by age. Younger children often require weight-based dosing, careful titration of topical steroids, and stricter safety margins to avoid adverse effects on growth and bone health, whereas adults may tolerate higher absolute doses but are more likely to have comorbidities and concomitant medications that influence efficacy and safety profiles [159]. The chronic nature of EoE means that treatments started in childhood may continue for decades, necessitating consideration of long-term safety and efficacy. Older adolescents in trials may transition to adult care during long-term studies, creating continuity challenges. Innovative pediatric trial designs, pediatric-specific outcome measures, and strategies for long-term follow-up are needed to advance this field.

### 3.9. Future Therapeutic Directions

Enhancing drug delivery and patient adherence is a key focus for EoE therapies. Current topical steroid regimens often demand daily or twice-daily dosing over long durations, which can strain patient compliance [160]. Pharmacokinetic limits, such as rapid esophageal clearance, necessitate measures like post-dose fasting to prolong mucosal contact [160]. Indeed, studies confirm that every-other-day dosing fails to maintain histologic remission in youth, underscoring the need for daily treatment to sustain drug exposure [160]. Not surprisingly, objective monitoring has revealed suboptimal adherence to swallowed steroid therapy, due in part to the burden of frequent, unpalatable dosing and difficulties for pediatric or dysphagic patients [161].

Recent advances in formulation science aim to mitigate these issues. A budesonide ODT has demonstrated robust efficacy in inducing and maintaining remission, providing a standardized, palatable alternative to extemporaneously prepared oral viscous suspensions [160]. Likewise, a fluticasone ODT is in late-phase trials, and newer topical steroids such as mometasone furoate show promise with once-daily dosing, made feasible by high topical potency and low systemic bioavailability [57]. Novel drug-device combinations are also being explored to prolong esophageal drug residence. For instance, a swallowable capsule (EsoCap) can release a mucoadhesive film that adheres to the esophagus, enabling sustained drug release and segment-specific targeting of medication [162]. Similarly, an esophageal corticosteroid-eluting string (swallowed and left in place overnight) and a 3D-printed drug-releasing ring have achieved high local steroid levels in preclinical models, lasting 1–3 days with minimal systemic absorption [163]. Such approaches could drastically reduce dosing frequency while maximizing mucosal contact time.

The phase-II ACESO trial demonstrated that this thin mucoadhesive film loaded with mometasone (ESO-101) reduced peak eosinophil counts by ~49 per high-power field compared with placebo and induced histologic remission in nearly half of the treated adults, with no serious adverse events [60]. The film ensures prolonged mucosal contact without oral exposure, and the absorbed mometasone undergoes extensive first-pass metabolism, resulting in low systemic bioavailability [60]. Beyond films, stent-based, film-based and nanoparticle-based esophageal drug delivery systems are under exploration to improve mucosal retention time and permeability [164]. Emerging formulation innovations also include mucoadhesive liquid preparations; for example, hydroxypropyl-β-cyclodextrin/carboxymethylcellulose-based budesonide solutions have shown suitable rheology and mucoadhesive properties for prolonged esophageal residence [165]. Nanoparticle-assisted formulations are being investigated to deliver corticosteroids and biologics with controlled release and minimal systemic absorption [164].

Alternative delivery routes are also under consideration: subcutaneous biologic therapy administered weekly has produced significant histologic and symptomatic improvements in EoE, highlighting the potential of injectable long-acting treatments to bypass the esophagus completely [88]. In the future, transnasal or transmucosal delivery systems (e.g., esophageal sprays or adhesive oralmucosal films) might further facilitate administration for patients with severe swallowing difficulties [48].

Preclinical studies are evaluating immunomodulatory probiotics: supplementation with *Lactococcus lactis* NCC 2287 during treatment reduced esophageal eosinophilia and boosted IL-10 production, and Clostridia-based interventions or other strains may complement existing therapies [166]. These probiotics influence microRNA expression and hint at epigenetic effects [166]. Epigenetically, IL-13-induced eotaxin-3 expression requires histone H3 acetylation and DNA demethylation; proton-pump inhibitors have been shown in vitro to reduce H3K4 trimethylation and eotaxin-3 expression [167]. Dysregulated microRNAs—miR-21 and miR-223 (upregulated) and miR-375 (downregulated)—correlate with eosinophil burden and represent potential therapeutic targets [167].

Recently, eosinophil-derived neurotoxin (EDN) measured via Cytosponge or esophageal string tests differentiates active disease from remission with 97% sensitivity and 89% specificity and aligns closely with endoscopic and histologic scores [168]. Serum periostin, an IL-13–induced extracellular matrix protein, remains a candidate biomarker despite only modestly elevated baseline levels compared with controls [169]. Fractional exhaled nitric oxide alone lacks diagnostic precision, but combining FeNO with EDN and periostin may improve non-invasive monitoring [168].

In one case report, a teenager receiving dupilumab maintained remission only when a topical steroid was continued; stopping the steroid led to relapse, and restarting it restored remission [170]. This observation suggests that pairing biologics with optimized topical steroids could enhance and sustain therapeutic responses.

Finally, artificial-intelligence tools based on detecting subtle endoscopic signs and calculating the EREFS score with sensitivities and specificities above 90%, outperforming less-experienced endoscopists [171,172]. Integrating clinical, endoscopic, histologic and molecular data can help identify disease subtypes and predict dietary or pharmacologic responses [172].

From a clinical standpoint, we believe that the current evidence supports a pragmatic, stepwise approach in which optimized topical corticosteroids and high-dose PPI therapy remain the backbone of care for most patients, with biologics such as dupilumab reserved for individuals with refractory disease, prominent atopic multimorbidity, or high-risk fibrostenotic phenotypes. Looking ahead, however, the greatest incremental gains are unlikely to come from simply adding more cytokine-specific agents, but rather from integrating potent, mechanism-based drugs with smarter drug-delivery platforms, earlier intervention in well-defined endotypes, and treat-to-target strategies anchored to composite clinicopathologic outcomes. In our view, parallel priorities include comparative effectiveness studies of diet–drug combinations, longitudinal registries that capture EndoFLIP metrics, molecular biomarkers and patient-reported outcomes, and pediatric-focused programs to define life-course treatment strategies. Ultimately, we envisage a future landscape in which endotype-driven algorithms replace the current “one-size-fits-all” paradigm, using tailored combinations of dietary interventions, topical formulations, biologics and small molecules to prevent progression to fibrostenosis while minimizing treatment burden and healthcare costs.

## 4. Conclusions

EoE research requires innovative approaches due to disease heterogeneity, suggesting benefits from precision medicine based on molecular markers and disease mechanisms. Traditional clinical trial designs are inadequate; adaptive platform trials, SMART designs, and individualized approaches may better address EoE complexity. Recent trials reveal EoE is not just an “eosinophilic” disease but a complex immunologic disorder, as eosinophil depletion alone does not improve symptoms. Research must move beyond eosinophil counts to address multidimensional aspects, including inflammation, structural changes, and psychological factors. An integrated assessment framework should incorporate broader inflammatory markers, structural parameters, psychological evaluation, and prognostic biomarkers. Trials must bridge the gap between clinical research and real-world outcomes by addressing psychological comorbidities and improving treatment implementation. Collaboration among multidisciplinary teams, including gastroenterologists, allergists, pathologists, psychologists, and patient advocates, is essential. The impact of psychosocial factors on EoE outcomes necessitates a holistic approach addressing both biological and psychological dimensions. Disease modification is a critical research frontier, focusing on preventing progression to fibrostenotic complications through early intervention. Future research should develop precision medicine approaches matching specific interventions to individual patient characteristics across the diverse EoE spectrum.

## Figures and Tables

**Table 1:** Overview of phase 1 and early-phase investigational therapies and mechanistic studies in eosinophilic esophagitis.

Drug Type	Drug Name	Phase	Approved	Regulatory Status	Study Status (NCT)	Years	Age Range
**PHASE 1 AND EARLY PHASE STUDIES**							
Bioavailability study	AQ280	Phase 1	No	Bioavailability comparison of capsule vs. tablet formulations	Active, not recruiting (NCT07093008)	2025+	18–65 years
SAD/MAD/Food effect study	AQ280	Phase 1	No	Safety, tolerability, and pharmacokinetics completed	Completed WITH RESULTS (NCT05485779)	2022–2023	18–65 years
Orodispersible formulation	BT-11	Phase 1	No	Safety and pharmacokinetics study	Withdrawn (NCT04835168)	2022	18–65 years
Drug-drug interaction study	Cendakimab	Phase 1	No	Disease-mediated drug interactions	Completed (NCT05175352)	2022–2024	18–75 years
Mucosal protectant	Sucralfate	Early Phase 1	No	Effectiveness and mucosal impedance	Completed (NCT02353078)	2015–2016	18–80 years
Biomarker identification	Omalizumab	Phase 1	No	Identify responder markers	Completed (NCT01040598)	2009–2011	12–76 years
Inflammatory markers study	Beclomethasone dipropionate	Phase 1	No	Effect on inflammatory markers	Completed (NCT01016223)	2010–2012	18–65 years
Intestinal permeability	Absorbable sugars	Phase 1	No	GI permeability in response to steroids	Completed (NCT01641913)	2012–2014	18–80 years
Efficacy evaluation	Montelukast	Phase 1	No	Effectiveness and safety evaluation	Completed (NCT00511316)	2007–2015	18–100 years
Hypereosinophilic syndrome	SCH55700 (Anti-IL-5)	Phase 1/2	No	Early anti-IL-5 antibody study	Completed (NCT00017862)	2001–2003	All ages
Immunosuppressant	Sirolimus	Phase 1	No	Safety in eosinophil-associated GI disorders	Terminated (NCT01814059)	2013–2015	18–65 years

Abbreviations: SAD, single ascending dose; MAD, multiple ascending dose; GI, gastrointestinal; NCT, National Clinical Trial; IL, interleukin.

**Table 2:** Phase 2 and 3 clinical trials of topical corticosteroids and steroid-based formulations in eosinophilic esophagitis.

Drug Name	Phase, Approved	Regulatory Status	Study Status (NCT)	Years	Age Range
Budesonide ODT	3, yes	EMA approved (Europe 2018), Health Canada, TGA (Australia); Not USFDA approved	Recruiting (NCT06596252); Completed (NCT02434029, NCT02493335)	2016–2018, 2024+	18–75
Budesonide Oral Suspension (BOS)	3, yes	USFDA approved (2024)—First official oral treatment for EoE in US	Terminated (NCT03245840); Completed (NCT02605837)	2020–2024	11–55
Budesonide effervescent tablets	2, no	Dose-ranging study vs. viscous suspension	Completed (NCT02280616)	2011–2014	18–75
Budesonide viscous (pediatric)	2, no	Pediatric dose-ranging study	Completed WITH RESULTS (NCT00762073)	2009–2010	2–18
Budesonide + PPI	2, no	Combination with lansoprazole	Completed WITH RESULTS (NCT00638456)	2008–2009	1+
Budesonide (Swiss study)	2, no	Induction and maintenance study	Completed (NCT00271349)	2005–2008	14–70
Budesonide Oral Suspension	2, no	Early development study	Completed WITH RESULTS (NCT01642212)	2012–2014	11–40
Fluticasone propionate (early)	2, no	Early topical steroid study	Completed (NCT00275561)	2005–2010	18–60
Fluticasone propionate (pediatric)	3, no	Swallowed Flovent vs. placebo	Completed (NCT00266578)	2002–2012	3–30
Mometasone-furoate inhaler	2, no	Aerosolized steroid study	Terminated (NCT02113267)	2014–2018	18+
Florence Oral Suspension	2, no	Phase 2 recruiting	Recruiting (NCT02873468)	2016+	18+
Fluticasone ODT (APT-1011)	3, no	FLUTE 3 trial completed; awaiting regulatory submission	Completed (NCT05634746, NCT04281108); Phase 2 (NCT03191864)	2017, 2020–2022	18+
Injectable extended-release fluticasone (EP-104GI)	1/2, no	In development	Recruiting (NCT05608681)	2022+	18–75
Mometasone furoate (ESO-101)	2, no	Phase 2 completed; Phase 3 needed	Completed (NCT04849390)	2021–2023	18–70

Abbreviations: EoE, eosinophilic esophagitis; PPI, proton-pump inhibitor;

**Table 3:** Biologic and other targeted pharmacologic and dietary interventions in eosinophilic esophagitis: summary of clinical trials and comparative studies.

**ANTI-IL-5 PATHWAY**							
Anti-IL-5 monoclonal antibody	Mepolizumab	Phase 2	No	Development discontinued for EoE	Completed (NCT03656380, NCT00358449, NCT00274703)	2006–2010, 2018–2020	16–75 years
Anti-IL-5 monoclonal antibody	Reslizumab	Phase 2/3	No	Development discontinued for EoE	Completed (NCT00538434)	2012	5–18 years
Anti-IL-5Rα monoclonal antibody	Benralizumab	Phase 3	No	Development terminated for EoE	Terminated WITH RESULTS (NCT04543409)	2024	12–65 years
Early anti-IL-5 antibody	SCH55700	Phase 2	No	Early IL-5 inhibitor study completed	Completed (NCT00017862)	2006–2008	All ages
**ANTI-IL-4/IL-13 PATHWAY**							
Anti-IL-4Rα monoclonal antibody	Dupilumab	Phase 3	Yes	USFDA approved (2022), EMA approved—First biologic for EoE	Completed WITH RESULTS (NCT03633617, NCT04394351, NCT02379052)	2015–2018, 2019–2022	1+ years (pediatric), 12+ years (adult)
Anti-IL-13 monoclonal antibody	Cendakimab (CC-93538)	Phase 3	No	Phase 3 positive results: Significant improvements in symptoms and eosinophils sustained through 48 weeks; regulatory submission expected	Active not recruiting (NCT04991935); Completed WITH RESULTS (NCT04753697); Phase 2 (NCT02098473)	2024–2027; 2014–2019, 2022–2024	12–75 years
Anti-IL-13 monoclonal antibody	Dectrekumab (QAX576)	Phase 2	No	Development discontinued	Completed (NCT01022970)	2014	18–50 years
**ANTI-TSLP PATHWAY**							
Anti-TSLP monoclonal antibody	Tezepelumab	Phase 3	No	Phase 3 ongoing	Active, not recruiting (NCT05583227)	2023–2027	12–80 years
Anti-TSLP monoclonal antibody	Solrikitug	Phase 2	No	Phase 2 ongoing	Recruiting (NCT06598462)	2027+	18–75 years
**OTHER BIOLOGICS**							
Anti-Siglec-8 monoclonal antibody	Lirentelimab (AK002)	Phase 2/3	No	Development challenges due to mixed efficacy	Completed WITH RESULTS (NCT04322708)	2022	12–80 years
Anti-IgE monoclonal antibody	Omalizumab	Phase 2	No	Limited efficacy demonstrated	Completed WITH RESULTS (NCT00123630)	2008–2012	12–60 years
Anti-IL-15 monoclonal antibody	CALY-002	Phase 1a/1b	No	Early development; EU and US Orphan Drug Designation	Phase 1 ongoing	2024+	TBD
Anti-KIT monoclonal antibody	Barzolvolimab (CDX-0159)	Phase 2	No	Phase 2 results expected H2 2025	Active, not recruiting (NCT05774184)	2023–2025	18+ years
Anti-TNF monoclonal antibody	Infliximab	Phase 2	No	Phase 2 completed	Completed (NCT00523354)	2010–2013	18–70 years
**ACID SUPPRESSION/EOTAXIN 3 INHIBITION**							
PCAB (Potassium-competitive acid blocker)	Vonoprazan	Phase 2	No	Phase 2 not yet started	Not yet recruiting (NCT06851559)	2026+	12+ years
PPI	Esomeprazole	Phase 2/3	No	Multiple studies completed	Completed (NCT00123656, NCT00728481)	2008	18–80 years
PPI	Dexlansoprazole	Phase 1/2	No	Study withdrawn	Withdrawn (NCT01479231)	2012–2013	18–80 years
**OTHER THERAPIES**							
Anti-inflammatory (5-ASA)	Mesalazine	Phase 2	No	Phase 2 completed	Completed (NCT05488405)	2022–2024	18–75 years
Alpha-1 proteinase inhibitor	Zemaira	Phase 2	No	Phase 2 recruiting	Recruiting (NCT05485155)	2022+	18–70 years
H2 antagonist/Antihistamine	Famotidine + Loratadine	Phase 2	No	Development terminated	Terminated WITH RESULTS (NCT04248712)	2020–2021	18+ years
ARB (Angiotensin receptor blocker)	Losartan	Phase 2	No	Multiple Phase 2 trials completed	Completed WITH RESULTS (NCT03029091, NCT01808196)	2013–2018	5–25 years
Epicutaneous immunotherapy	Viaskin Milk Patch	Phase 2	No	Phase 2 completed	Completed WITH RESULTS (NCT02579876)	2016–2019	4–17 years
Cholinergic agonist	Bethanechol	Phase 2	No	Development terminated	Terminated WITH RESULTS (NCT02058537)	2014–2016	18–75 years
Leukotriene receptor antagonist	Montelukast	Phase 3	No	Study withdrawn	Withdrawn (NCT01702701)	2012	18+ years
Anti JAK1	AQ280	Phase 1	No	Novel mechanism (undisclosed) in development	Phase 1 studies completed and ongoing (NCT05485779, NCT07093008)	2022+	18–65 years
Mycobacterium tuberculosis peptide	IRL201104	Phase 2	No	Phase 2b planned; USFDA Orphan Drug Designation	Completed WITH RESULTS (NCT05084963)—modest efficacy: 8 mg dose showed −31.6 eos/hpf reduction vs. −7.8 placebo	2022–2025	18–75 years
S1P 1/4/5 modulator (oral)	Etrasimod (APD334)	Phase 2b	No	Development discontinued despite positive Phase 2	Completed WITH RESULTS (NCT04682639)	2023	18–65 years
CRTH2/DP2 antagonist	OC000459 (Timapiprant)	Phase 2	No	Phase 2 completed	Completed (NCT01056783)	2012–2015	18–75 years
**COMPARISONS**							
Dupilumab vs. Topical steroid	Dupilumab vs. Fluticasone	Phase 2	No	Head-to-head comparison in stenotic EoE	Recruiting (NCT06705387)	2024–2027	12–25 years
Dietary intervention	Six-food vs. One-food elimination diet	Phase 2/3	No	SOFEED study comparing elimination diets	Completed WITH RESULTS (NCT02778867)	2016–2019	18–60 years
Dietary intervention	One-food vs. Four-food elimination diet	Phase 2/3	No	Pediatric diet comparison with glucocorticoids	Completed WITH RESULTS (NCT02610816)	2016–2018	6–17 years
Treatment comparison	Budesonide vs. Elimination diet	Phase 2	No	Direct therapy comparison	Terminated WITH RESULTS (NCT01821898)	2013–2018	3–17 years

Abbreviations: EoE, eosinophilic esophagitis; PPI, proton-pump inhibitor; TSLP, thymic stromal lymphopoietin; CRTH2/DP2, chemoattractant receptor–homologous molecule expressed on Th2 cells; S1P, sphingosine-1-phosphate; eos/hpf, eosinophils per high-power field.

## Data Availability

This review did not generate new data; data sharing is not applicable.

## Abbreviations

The following abbreviations are used in this manuscript:

AE	Adverse event
APC	Antigen-presenting cell
BET	Budesonide effervescent tablet
BID	Twice daily
BOS	Budesonide oral suspension
CCL26	Chemokine (eotaxin-3)
CRTH2/DP2	Chemoattractant receptor-homologous molecule expressed on Th2 cells/prostaglandin D2 receptor 2
DSQ	Dysphagia Symptom Questionnaire
EGJ	Esophagogastric junction
EMA	European Medicines Agency
EoE	Eosinophilic esophagitis
EoEHSS	EoE Histology Scoring System
EEsAI	EoE Activity Index
EREFS	EoE Endoscopic Reference Score
USFDA	U.S. Food and Drug Administration
FLIP	Functional lumen imaging probe
HPF/eos/hpf	High-power field/eosinophils per high-power field
HR	Histologic remission
IgE	Immunoglobulin E
IL	Interleukin (e.g., IL-4, IL-5, IL-13, IL-15)
ILC2	Type-2 innate lymphoid cell
JAK	STAT-Janus kinase-signal transducer and activator of transcription
KIT	Stem cell factor receptor (CD117)
mAb	Monoclonal antibody
ODT	Orally disintegrating tablet
OLE	Open-label extension
P-CAB (PCAB)	Potassium-competitive acid blocker
PPI	Proton-pump inhibitor
PRO	Patient-reported outcome
RCT	Randomized controlled trial
RDBPC	Randomized double-blind placebo-controlled
SAE	Serious adverse event
SC/IV/PO	Subcutaneous/Intravenous/Oral
Siglec-8	Sialic-acid-binding immunoglobulin-like lectin-8
S1P	Sphingosine-1-phosphate
STC	Swallowed topical corticosteroid
Th2	Type-2 helper T cell
TNF	Tumor necrosis factor
TSLP	Thymic stromal lymphopoietin

## References

[1] de Bortoli N., Visaggi P., Penagini R., Annibale B., Baiano Svizzero F., Barbara G., Bartolo O., Battaglia E., Di Sabatino A., De Angelis P. (2024). The 1st EoETALY Consensus on the Diagnosis and Management of Eosinophilic Esophagitis-Current Treatment and Monitoring. Dig. Liver Dis. Off. J. Ital. Soc. Gastroenterol. Ital. Assoc. Study Liver.

[2] Pouw R.E., Barret M., Biermann K., Bisschops R., Czakó L., Gecse K.B., de Hertogh G., Hucl T., Iacucci M., Jansen M. (2021). Endoscopic Tissue Sampling—Part 1: Upper Gastrointestinal and Hepatopancreatobiliary Tracts. European Society of Gastrointestinal Endoscopy (ESGE) Guideline. Endoscopy.

[3] Lucendo A.J., Santander C., Savarino E., Guagnozzi D., Pérez-Martínez I., Perelló A., Guardiola-Arévalo A., Barrio J., Elena Betoré-Glaria M., Gutiérrez-Junquera C. (2022). EoE CONNECT, the European Registry of Clinical, Environmental, and Genetic Determinants in Eosinophilic Esophagitis: Rationale, Design, and Study Protocol of a Large-Scale Epidemiological Study in Europe. Ther. Adv. Gastroenterol..

[4] Mari A., Calabrese F., Pasta A., Lorenzon G., Weusten B., Keller J., Visaggi P., Roman S., Marabotto E., Dickman R. (2025). Esophageal and Oropharyngeal Dysphagia: Clinical Recommendations From the United European Gastroenterology and European Society for Neurogastroenterology and Motility. United Eur. Gastroenterol. J..

[5] Dellon E.S., Hirano I. (2018). Epidemiology and Natural History of Eosinophilic Esophagitis. Gastroenterology.

[6] Muir A., Falk G.W. (2021). Eosinophilic Esophagitis: A Review. JAMA.

[7] de Bortoli N., Visaggi P., Penagini R., Annibale B., Baiano Svizzero F., Barbara G., Bartolo O., Battaglia E., Di Sabatino A., De Angelis P. (2024). The 1st EoETALY Consensus on the Diagnosis and Management of Eosinophilic Esophagitis—Definition, Clinical Presentation and Diagnosis. Dig. Liver Dis. Off. J. Ital. Soc. Gastroenterol. Ital. Assoc. Study Liver.

[8] Ryu S., Lee K.H., Tizaoui K., Terrazzino S., Cargnin S., Effenberger M., Shin J.I., Kronbichler A. (2020). Pathogenesis of Eosinophilic Esophagitis: A Comprehensive Review of the Genetic and Molecular Aspects. Int. J. Mol. Sci..

[9] Visaggi P., Ghisa M., Barberio B., Marabotto E., de Bortoli N., Savarino E. (2022). Systematic Review: Esophageal Motility Patterns in Patients with Eosinophilic Esophagitis. Dig. Liver Dis..

[10] Visaggi P., Ghisa M., Marabotto E., Venturini A., Stefani Donati D., Bellini M., Savarino V., de Bortoli N., Savarino E. (2023). Esophageal Dysmotility in Patients with Eosinophilic Esophagitis: Pathogenesis, Assessment Tools, Manometric Characteristics, and Clinical Implications. Esophagus.

[11] Ghisa M., Laserra G., Marabotto E., Ziola S., Tolone S., de Bortoli N., Frazzoni M., Mauro A., Penagini R., Savarino V. (2021). Achalasia and Obstructive Motor Disorders Are Not Uncommon in Patients with Eosinophilic Esophagitis. Clin. Gastroenterol. Hepatol..

[12] Racca F., Pellegatta G., Cataldo G., Vespa E., Carlani E., Pelaia C., Paoletti G., Messina M.R., Nappi E., Canonica G.W. (2021). Type 2 Inflammation in Eosinophilic Esophagitis: From Pathophysiology to Therapeutic Targets. Front. Physiol..

[13] Chehade M., Falk G.W., Aceves S., Lee J.K., Mehta V., Leung J., Shumel B., Jacob-Nara J.A., Deniz Y., Rowe P.J. (2022). Examining the Role of Type 2 Inflammation in Eosinophilic Esophagitis. Gastro Hep Adv..

[14] Doyle A.D., Masuda M.Y., Kita H., Wright B.L. (2020). Eosinophils in Eosinophilic Esophagitis: The Road to Fibrostenosis Is Paved with Good Intentions. Front. Immunol..

[15] Simon D., Wardlaw A., Rothenberg M.E. (2010). Organ-Specific Eosinophilic Disorders of the Skin, Lung, and Gastrointestinal Tract. J. Allergy Clin. Immunol..

[16] Martin L.J., He H., Collins M.H., Abonia J.P., Biagini Myers J.M., Eby M., Johansson H., Kottyan L.C., Khurana Hershey G.K., Rothenberg M.E. (2018). Eosinophilic Esophagitis (EoE) Genetic Susceptibility Is Mediated by Synergistic Interactions between EoE-Specific and General Atopic Disease Loci. J. Allergy Clin. Immunol..

[17] Hahn J.W., Lee K., Shin J.I., Cho S.H., Turner S., Shin J.U., Yeniova A.Ö., Koyanagi A., Jacob L., Smith L. (2023). Global Incidence and Prevalence of Eosinophilic Esophagitis, 1976–2022: A Systematic Review and Meta-Analysis. Clin. Gastroenterol. Hepatol. Off. Clin. Pract. J. Am. Gastroenterol. Assoc..

[18] Thel H.L., Anderson C., Xue A.Z., Jensen E.T., Dellon E.S. (2025). Prevalence and Costs of Eosinophilic Esophagitis in the United States. Clin. Gastroenterol. Hepatol..

[19] Dellon E.S., Liacouras C.A., Molina-Infante J., Furuta G.T., Spergel J.M., Zevit N., Spechler S.J., Attwood S.E., Straumann A., Aceves S.S. (2018). Updated International Consensus Diagnostic Criteria for Eosinophilic Esophagitis: Proceedings of the AGREE Conference. Gastroenterology.

[20] Feo-Ortega S., Lucendo A.J. (2022). Evidence-Based Treatments for Eosinophilic Esophagitis: Insights for the Clinician. Ther. Adv. Gastroenterol..

[21] Runge T.M., Eluri S., Cotton C.C., Burk C.M., Woosley J.T., Shaheen N.J., Dellon E.S. (2016). Outcomes of Esophageal Dilation in Eosinophilic Esophagitis: Safety, Efficacy, and Persistence of the Fibrostenotic Phenotype. Am. J. Gastroenterol..

[22] Farah A., Assaf T., Hindy J., Abboud W., Mahamid M., Savarino E.V., Mari A. (2025). The Dynamic Evolution of Eosinophilic Esophagitis. Diagnostics.

[23] Facchin S., Bonazzi E., Tomasulo A., Bertin L., Lorenzon G., Maniero D., Zingone F., Cardin R., Barberio B., Ghisa M. (2025). Could Modulating the Esophageal Microbiome Be the Answer for Eosinophilic Esophagitis Treatment?. Expert Rev. Gastroenterol. Hepatol..

[24] Visaggi P., Savarino E., Del Corso G., Hunter H., Baiano Svizzero F., Till S.J., Dunn J., Wong T., de Bortoli N., Zeki S. (2023). Six-Food Elimination Diet Is Less Effective During Pollen Season in Adults with Eosinophilic Esophagitis Sensitized to Pollens. Am. J. Gastroenterol..

[25] Visaggi P., Baiano Svizzero F., Savarino E. (2023). Food Elimination Diets in Eosinophilic Esophagitis: Practical Tips in Current Management and Future Directions. Best Pract. Res. Clin. Gastroenterol..

[26] Molina-Infante J., Arias Á., Alcedo J., Garcia-Romero R., Casabona-Frances S., Prieto-Garcia A., Modolell I., Gonzalez-Cordero P.L., Perez-Martinez I., Martin-Lorente J.L. (2018). Step-up Empiric Elimination Diet for Pediatric and Adult Eosinophilic Esophagitis: The 2-4-6 Study. J. Allergy Clin. Immunol..

[27] Pasta A., Calabrese F., Furnari M., Savarino E.V., Visaggi P., Bodini G., Formisano E., Zentilin P., Giannini E.G., Marabotto E. (2025). Endoscopic Management of Eosinophilic Esophagitis: A Narrative Review on Diagnosis and Treatment. J. Clin. Med..

[28] Lucendo A.J., Arias Á., González-Cervera J., Yagüe-Compadre J.L., Guagnozzi D., Angueira T., Jiménez-Contreras S., González-Castillo S., Rodríguez-Domíngez B., De Rezende L.C. (2013). Empiric 6-Food Elimination Diet Induced and Maintained Prolonged Remission in Patients with Adult Eosinophilic Esophagitis: A Prospective Study on the Food Cause of the Disease. J. Allergy Clin. Immunol..

[29] Kagalwalla A.F., Wechsler J.B., Amsden K., Schwartz S., Makhija M., Olive A., Davis C.M., Manuel-Rubio M., Marcus S., Shaykin R. (2017). Efficacy of a 4-Food Elimination Diet for Children with Eosinophilic Esophagitis. Clin. Gastroenterol. Hepatol. Off. Clin. Pract. J. Am. Gastroenterol. Assoc..

[30] Molina-Infante J., Gonzalez-Cordero P.L., Arias A., Lucendo A.J. (2017). Update on Dietary Therapy for Eosinophilic Esophagitis in Children and Adults. Expert Rev. Gastroenterol. Hepatol..

[31] Kliewer K.L., Gonsalves N., Dellon E.S., Katzka D.A., Abonia J.P., Aceves S.S., Arva N.C., Besse J.A., Bonis P.A., Caldwell J.M. (2023). One-Food versus Six-Food Elimination Diet Therapy for the Treatment of Eosinophilic Oesophagitis: A Multicentre, Randomised, Open-Label Trial. lancet. Gastroenterol. Hepatol..

[32] Mayerhofer C., Kavallar A.M., Aldrian D., Lindner A.K., Müller T., Vogel G.F. (2023). Efficacy of Elimination Diets in Eosinophilic Esophagitis: A Systematic Review and Meta-Analysis. Clin. Gastroenterol. Hepatol. Off. Clin. Pract. J. Am. Gastroenterol. Assoc..

[33] Warners M.J., Vlieg-Boerstra B.J., Verheij J., van Rhijn B.D., Van Ampting M.T.J., Harthoorn L.F., de Jonge W.J., Smout A.J.P.M., Bredenoord A.J. (2017). Elemental Diet Decreases Inflammation and Improves Symptoms in Adult Eosinophilic Oesophagitis Patients. Aliment. Pharmacol. Ther..

[34] Marabotto E., Pasta A., Calabrese F., Ribolsi M., Mari A., Savarino V., Savarino E.V. (2024). The Clinical Spectrum of Gastroesophageal Reflux Disease: Facts and Fictions. Visc. Med..

[35] Navarro P., Laserna-Mendieta E.J., Guagnozzi D., Casabona S., Perelló A., Savarino E., de la Riva S., Olalla J.M., Ghisa M., Serrano-Moya N. (2021). Proton Pump Inhibitor Therapy Reverses Endoscopic Features of Fibrosis in Eosinophilic Esophagitis. Dig. Liver Dis..

[36] Frazzoni M., Frazzoni L., De Bortoli N., Russo S., Tolone S., Arsiè E., Conigliaro R., Penagini R., Savarino E. (2021). Response of Eosinophilic Oesophagitis to Proton Pump Inhibitors Is Associated with Impedance-pH Parameters Implying Anti-Reflux Mechanism of Action. Aliment. Pharmacol. Ther..

[37] Frazzoni M., Penagini R., Frazzoni L., de Bortoli N., Mauro A., Tolone S., Bertani H., Marsico M., Marocchi M., Marchi S. (2019). Role of Reflux in the Pathogenesis of Eosinophilic Esophagitis: Comprehensive Appraisal with off- and on PPI Impedance-pH Monitoring. Am. J. Gastroenterol..

[38] Visaggi P., Barberio B., Del Corso G., de Bortoli N., Black C.J., Ford A.C., Savarino E. (2023). Comparison of Drugs for Active Eosinophilic Oesophagitis: Systematic Review and Network Meta-Analysis. Gut.

[39] Visaggi P., Ghisa M., Vespa E., Barchi A., Mari A., Pasta A., Marabotto E., de Bortoli N., Savarino E.V. (2024). Optimal Assessment, Treatment, and Monitoring of Adults with Eosinophilic Esophagitis: Strategies to Improve Outcomes. Immuno Targets Ther..

[40] Meek P.D., Hemstreet B. (2023). Emerging Therapies for Eosinophilic Esophagitis. Pharmacother. J. Hum. Pharmacol. Drug Ther..

[41] Yang E.-J., Jung K.W. (2025). Role of Endoscopy in Eosinophilic Esophagitis. Clin. Endosc..

[42] Dellon E.S., Muir A.B., Katzka D.A., Shah S.C., Sauer B.G., Aceves S.S., Furuta G.T., Gonsalves N., Hirano I. (2025). ACG Clinical Guideline: Diagnosis and Management of Eosinophilic Esophagitis. Am. J. Gastroenterol..

[43] Chehade M., Hiremath G.S., Zevit N., Oliva S., Pela T., Khodzhayev A., Jacob-Nara J., Radwan A. (2024). Disease Burden and Spectrum of Symptoms That Impact Quality of Life in Pediatric Patients with Eosinophilic Esophagitis. Gastro Hep Adv..

[44] Al-Horani R.A., Chiles R. (2022). First Therapeutic Approval for Eosinophilic Esophagitis. Gastroenterol. Insights.

[45] Andreae D.A., Hanna M.G., Magid M.S., Malerba S., Andreae M.H., Bagiella E., Chehade M. (2016). Swallowed Fluticasone Propionate Is an Effective Long-Term Maintenance Therapy for Children with Eosinophilic Esophagitis. Am. J. Gastroenterol..

[46] Krishna S.G., Kakati B.R., Olden K.W., Brown D.K. (2011). Treatment of Eosinophilic Esophagitis. Gastroenterol. Hepatol..

[47] González-Cervera J., Lucendo A.J. (2016). Eosinophilic Esophagitis: An Evidence-Based Approach to Therapy. J. Investig. Allergol. Clin. Immunol..

[48] Barchi A., Girelli M., Ventimiglia A., Mandarino F.V., Danese S., Passaretti S., Yacoub M.-R., Nannipieri S., Ciliberto A.F., Albarello L. (2025). Orally Dispersible Swallowed Topical Corticosteroids in Eosinophilic Esophagitis: A Paradigm Shift in the Management of Esophageal Inflammation. Pharmaceutics.

[49] Gupta S.K., Hill M., Vitanza J.M., Farber R.H., Desai N.K., Williams J., Song I.H. (2022). Pharmacokinetics of Budesonide Oral Suspension in Children and Adolescents with Eosinophilic Esophagitis. J. Pediatr. Gastroenterol. Nutr..

[50] Lucendo A.J., Miehlke S., Schlag C., Vieth M., von Arnim U., Molina-Infante J., Hartmann D., Bredenoord A.J., Ciriza de Los Rios C., Schubert S. (2019). Efficacy of Budesonide Orodispersible Tablets as Induction Therapy for Eosinophilic Esophagitis in a Randomized Placebo-Controlled Trial. Gastroenterology.

[51] Straumann A., Lucendo A.J., Miehlke S., Vieth M., Schlag C., Biedermann L., Vaquero C.S., Ciriza de Los Rios C., Schmoecker C., Madisch A. (2020). Budesonide Orodispersible Tablets Maintain Remission in a Randomized, Placebo-Controlled Trial of Patients with Eosinophilic Esophagitis. Gastroenterology.

[52] Jorveza|European Medicines Agency (EMA). https://www.ema.europa.eu/en/medicines/human/EPAR/jorveza.

[53] Schoepfer A.M., Safroneeva E. (2024). Pharmacologic Treatment of Eosinophilic Esophagitis: Efficacious, Likely Efficacious, and Failed Drugs. Inflamm. *Intest.* Dis..

[54] Maniero D., Ghisa M., Bruschi A., Lorenzon G., Bertin L., Giorgini G., Bendia E., Coletta M., Penagini R., Visaggi P. (2025). Effectiveness and Safety of Orodispersible Budesonide for Eosinophilic Esophagitis: A Multicenter Real-World Study. Clin. Gastroenterol. Hepatol.

[55] Hirano I., Collins M.H., Katzka D.A., Mukkada V.A., Falk G.W., Morey R., Desai N.K., Lan L., Williams J., Dellon E.S. (2022). Budesonide Oral Suspension Improves Outcomes in Patients with Eosinophilic Esophagitis: Results from a Phase 3 Trial. Clin. Gastroenterol. Hepatol..

[56] FDA Approves EOHILIA (Budesonide Oral Suspension)|Takeda News. https://www.takeda.com/newsroom/newsreleases/2024/fda-approves-eohilia/.

[57] Dellon E.S., Lucendo A.J., Schlag C., Schoepfer A.M., Falk G.W., Eagle G., Nezamis J., Comer G.M., Knoop K., Hirano I. (2022). Fluticasone Propionate Orally Disintegrating Tablet (APT-1011) for Eosinophilic Esophagitis: Randomized Controlled Trial. Clin. Gastroenterol. Hepatol..

[58] (2022). 24-Week Induction Study of APT-1011 in Adult Subjects with Eosinophilic Esophagitis (EoE) (FLUTE-3).

[59] Study Details|NCT05608681|A Trial to Evaluate EP-104GI in Adults with Eosinophilic Esophagitis (EoE)|ClinicalTrials.gov. (n.d.). NCT05608681.

[60] Lucendo A.J., Nantes-Castillejo Ó., Straumann A., Biedermann L., Bredenoord A.J., Guagnozzi D., Blas-Jhon L., Wiechowska-Kozlowska A., Weidlich S., von Arnim U. (2025). Clinical Trial: Safety and Efficacy of a Novel Oesophageal Delivery System for Topical Corticosteroids Versus Placebo in the Treatment of Eosinophilic Oesophagitis. Aliment. Pharmacol. Ther..

[61] NCT04849390|A Study to Investigate the Efficacy and Tolerability of ESO-101 in Patients with Eosinophilic Esophagitis|ClinicalTrials.gov. (n.d.). NCT04849390.

[62] Tytor J., Larsson H., Bove M., Johansson L., Bergquist H. (2021). Topically Applied Mometasone Furoate Improves Dysphagia in Adult Eosinophilic Esophagitis—Results from a Double-Blind, Randomized, Placebo-Controlled Trial. Scand. J. Gastroenterol..

[63] (2016). Efficacy and Safety of Three Doses of Florence Oral Suspension in Adults with Eosinophilic Esophagitis (EMS0718–FLORENCE).

[64] Eupraxia Pharmaceuticals Inc (2025). Eupraxia Pharmaceuticals Announces Positive Data from Highest-Dose Cohort in the Ongoing RESOLVE Trial in Eosinophilic Esophagitis, and Plans for Expansion of EP-104GI Development Programs. https://eupraxiapharma.gcs-web.com/news-releases/news-release-details/eupraxia-pharmaceuticals-announces-positive-data-highest-dose.

[65] Ma J., Altomare A., Guarino M., Cicala M., Rieder F., Fiocchi C., Li D., Cao W., Behar J., Biancani P. (2012). HCl-Induced and ATP-Dependent Upregulation of TRPV1 Receptor Expression and Cytokine Production by Human Esophageal Epithelial Cells. Am. J. Physiol. Gastrointest. Liver Physiol..

[66] Kubo K., Kimura N. (2024). Long-Term Follow-up of Patients Developing Gastric Mucosal Lesions after Initiating the Potassium-Competitive Acid Blocker Vonoprazan. Clin. Endosc..

[67] Marabotto E., Calabrese F., Pasta A., Visaggi P., de Bortoli N., Mari A., Tolone S., Ghisa M., Bertin L., Savarino V. (2024). Evaluating Vonoprazan for the Treatment of Erosive GERD and Heartburn Associated with GERD in Adults. Expert Opin. Pharmacother..

[68] Kuzumoto T., Tanaka F., Sawada A., Nadatani Y., Otani K., Hosomi S., Kamata N., Taira K., Nagami Y., Tanigawa T. (2021). Vonoprazan Shows Efficacy Similar to That of Proton Pump Inhibitors with Respect to Symptomatic, Endoscopic, and Histological Responses in Patients with Eosinophilic Esophagitis. Esophagus.

[69] Sawada A., Ihara Y., Imai T., Tanaka F., Fujiwara Y. (2024). Real World Treatment Patterns in Patients with Eosinophilic Esophagitis in Japan. Sci. Rep..

[70] Phathom Pharmaceuticals, Inc (2025). A Phase 2, Randomized, Double-Blind, Multi-Center, 3-Part Study in Adult and Adolescent Subjects with Eosinophilic Esophagitis (EoE) to Evaluate the Safety and Efficacy of Vonoprazan 10 mg and 20 mg Compared to Placebo After 12 Weeks and to Evaluate the Safety and Efficacy of Vonoprazan 10 mg and 20 mg Up to 52 Week.

[71] Massironi S., Mulinacci G., Gallo C., Elvevi A., Danese S., Invernizzi P., Vespa E. (2023). Mechanistic Insights into Eosinophilic Esophagitis: Therapies Targeting Pathophysiological Mechanisms. Cells.

[72] Uchida A.M., Burk C.M., Rothenberg M.E., Furuta G.T., Spergel J.M. (2023). Recent Advances in the Treatment of Eosinophilic Esophagitis. J. Allergy Clin. Immunol. Pract..

[73] Lombardi C., Comberiati P., Ridolo E., Cottini M., Yacoub M.R., Casagrande S., Riccò M., Bottazzoli M., Berti A. (2024). Anti-IL-5 Pathway Agents in Eosinophilic-Associated Disorders Across the Lifespan. Drugs.

[74] Straumann A., Conus S., Grzonka P., Kita H., Kephart G., Bussmann C., Beglinger C., Smith D.A., Patel J., Byrne M. (2010). Anti-Interleukin-5 Antibody Treatment (Mepolizumab) in Active Eosinophilic Oesophagitis: A Randomised, Placebo-Controlled, Double-Blind Trial. Gut.

[75] Assa’ad A.H., Gupta S.K., Collins M.H., Thomson M., Heath A.T., Smith D.A., Perschy T.L., Jurgensen C.H., Ortega H.G., Aceves S.S. (2011). An Antibody against IL-5 Reduces Numbers of Esophageal Intraepithelial Eosinophils in Children with Eosinophilic Esophagitis. Gastroenterology.

[76] Dellon E.S., Peterson K.A., Mitlyng B.L., Iuga A., Bookhout C.E., Cortright L.M., Walker K.B., Gee T.S., McGee S.J., Cameron B.A. (2023). Mepolizumab for Treatment of Adolescents and Adults with Eosinophilic Oesophagitis: A Multicentre, Randomised, Double-Blind, Placebo-Controlled Clinical Trial. Gut.

[77] Spergel J.M., Rothenberg M.E., Collins M.H., Furuta G.T., Markowitz J.E., Fuchs G., O’Gorman M.A., Abonia J.P., Young J., Henkel T. (2012). Reslizumab in Children and Adolescents with Eosinophilic Esophagitis: Results of a Double-Blind, Randomized, Placebo-Controlled Trial. J. Allergy Clin. Immunol..

[78] Markowitz J.E., Jobe L., Miller M., Frost C., Laney Z., Eke R. (2018). Safety and Efficacy of Reslizumab for Children and Adolescents with Eosinophilic Esophagitis Treated for 9 Years. J. Pediatr. Gastroenterol. Nutr..

[79] Hassani M., Koenderman L. (2018). Immunological and Hematological Effects of IL-5(Rα)-Targeted Therapy: An Overview. Allergy.

[80] Rothenberg M.E., Dellon E.S., Collins M.H., Bredenoord A.J., Hirano I., Peterson K.A., Brooks L., Caldwell J.M., Fjällbrant H., Grindebacke H. (2024). Eosinophil Depletion with Benralizumab for Eosinophilic Esophagitis. N. Engl. J. Med..

[81] A Study of Benralizumab in Patients with Eosinophilic Esophagitis. https://www.astrazenecaclinicaltrials.com/study/D3255C00001/.

[82] Le Floc’h A., Allinne J., Nagashima K., Scott G., Birchard D., Asrat S., Bai Y., Lim W.K., Martin J., Huang T. (2020). Dual Blockade of IL-4 and IL-13 with Dupilumab, an IL-4Rα Antibody, Is Required to Broadly Inhibit Type 2 Inflammation. Allergy.

[83] Colque-Bayona M., Hernández-Cano N., Tomás-Pérez M., Caballero T., Quirce S., Domínguez-Ortega J. (2024). Global Influence of Dupilumab on Quality of Life in a Severe Asthma Patient with T2 Multimorbidities: A Case Report on Atopic Dermatitis, Chronic Rhinosinusitis with Nasal Polyposis, and Eosinophilic Esophagitis. J. Asthma.

[84] Simpson E.L., Bieber T., Guttman-Yassky E., Beck L.A., Blauvelt A., Cork M.J., Silverberg J.I., Deleuran M., Kataoka Y., Lacour J.-P. (2016). Two Phase 3 Trials of Dupilumab versus Placebo in Atopic Dermatitis. N. Engl. J. Med..

[85] Bachert C., Han J.K., Desrosiers M., Hellings P.W., Amin N., Lee S.E., Mullol J., Greos L.S., Bosso J.V., Laidlaw T.M. (2019). Efficacy and Safety of Dupilumab in Patients with Severe Chronic Rhinosinusitis with Nasal Polyps (LIBERTY NP SINUS-24 and LIBERTY NP SINUS-52): Results from Two Multicentre, Randomised, Double-Blind, Placebo-Controlled, Parallel-Group Phase 3 Trials. Lancet.

[86] Hirano I., Dellon E.S., Hamilton J.D., Collins M.H., Peterson K., Chehade M., Schoepfer A.M., Safroneeva E., Rothenberg M.E., Falk G.W. (2020). Efficacy of Dupilumab in a Phase 2 Randomized Trial of Adults with Active Eosinophilic Esophagitis. Gastroenterology.

[87] Rothenberg M.E., Dellon E.S., Collins M.H., Hirano I., Chehade M., Bredenoord A.J., Lucendo A.J., Spergel J.M., Sun X., Hamilton J.D. (2023). Efficacy and Safety of Dupilumab up to 52 Weeks in Adults and Adolescents with Eosinophilic Oesophagitis (LIBERTY EoE TREET Study): A Multicentre, Double-Blind, Randomised, Placebo-Controlled, Phase 3 Trial. Lancet Gastroenterol. Hepatol..

[88] Dellon E.S., Rothenberg M.E., Collins M.H., Hirano I., Chehade M., Bredenoord A.J., Lucendo A.J., Spergel J.M., Aceves S., Sun X. (2022). Dupilumab in Adults and Adolescents with Eosinophilic Esophagitis. N. Engl. J. Med..

[89] Hirano I., Collins M.H., Assouline-Dayan Y., Evans L., Gupta S., Schoepfer A.M., Straumann A., Safroneeva E., Grimm M., Smith H. (2019). RPC4046, a Monoclonal Antibody Against IL13, Reduces Histologic and Endoscopic Activity in Patients with Eosinophilic Esophagitis. Gastroenterology.

[90] (2021). A Phase 3, Multicenter, Multinational, Randomized, Double-Blind, Placebo-Controlled Induction and Maintenance Study to Evaluate the Efficacy and Safety of CC-93538 in Adult and Adolescent Subjects with Eosinophilic Esophagitis (CC-93538-EE-001).

[91] Rothenberg M.E., Wen T., Greenberg A., Alpan O., Enav B., Hirano I., Nadeau K., Kaiser S., Peters T., Perez A. (2015). Intravenous Anti-IL-13 mAb QAX576 for the Treatment of Eosinophilic Esophagitis. J. Allergy Clin. Immunol..

[92] Hirano I., Dellon E.S., Falk G.W., Gonsalves N.P., Furuta G.T., Bredenoord A.J., Ascent Working Group (2024). Ascending to New Heights for Novel Therapeutics for Eosinophilic Esophagitis. Gastroenterology.

[93] Sindher S.B., Barshow S., Tirumalasetty J., Arasi S., Atkins D., Bauer M., Bégin P., Collins M.H., Deschildre A., Doyle A.D. (2023). The Role of Biologics in Pediatric Food Allergy and Eosinophilic Gastrointestinal Disorders. J. Allergy Clin. Immunol..

[94] NCT05583227. NCT05583227.

[95] Sharlin C.S., Collins M.H., Bolton S.M., Osswald G.A., Safadi G.S., Kliewer K.L., Rothenberg M.E., Shoda T., Mukkada V.A. (2024). Induction of Sustained Remission and Reversal of Pathologic Transcriptome Achieved with Tezepelumab in an Adolescent with Eosinophilic Esophagitis. J. Allergy Clin. Immunol. Pract..

[96] Uniquity One (UNI) (2024). A Phase 2, Randomized, Double-Blind, Multicenter, Placebo Controlled Study with an Open Label Extension to Investigate the Efficacy and Safety of NSI-8226 in Adults with Eosinophilic Esophagitis (ALAMERE) (NSI-8226-201).

[97] Bochner B.S. (2009). Siglec-8 on Human Eosinophils and Mast Cells, and Siglec-F on Murine Eosinophils, Are Functionally Related Inhibitory Receptors. Clin. Exp. Allergy.

[98] Schanin J., Gebremeskel S., Korver W., Falahati R., Butuci M., Haw T.J., Nair P.M., Liu G., Hansbro N.G., Hansbro P.M. (2021). A Monoclonal Antibody to Siglec-8 Suppresses Non-Allergic Airway Inflammation and Inhibits IgE-Independent Mast Cell Activation. Mucosal Immunol..

[99] (2023). A Phase 2/3, Multicenter, Randomized, Double-Blind, Placebo-Controlled Study to Evaluate the Efficacy and Safety of Lirentelimab (AK002) in Adult and Adolescent Patients with Active Eosinophilic Esophagitis (KRYPTOS).

[100] Dellon E., Chehade M., Genta R.M., Leiman D.A., Peterson K.A., Spergel J., Wechsler J., Bortey E., Chang A.T., Hirano I. (2022). S446 Results from KRYPTOS, a Phase 2/3 Study of Lirentelimab (AK002) in Adults and Adolescents with EoE. Off. J. Am. Coll. Gastroenterol. ACG.

[101] Loizou D., Enav B., Komlodi-Pasztor E., Hider P., Kim-Chang J., Noonan L., Taber T., Kaushal S., Limgala R., Brown M. (2015). A Pilot Study of Omalizumab in Eosinophilic Esophagitis. PLoS ONE.

[102] Clayton F., Fang J.C., Gleich G.J., Lucendo A.J., Olalla J.M., Vinson L.A., Lowichik A., Chen X., Emerson L., Cox K. (2014). Eosinophilic Esophagitis in Adults Is Associated with IgG4 and Not Mediated by IgE. Gastroenterology.

[103] Alba J.D., Ravanetti L., Corrigall V., Eggleton P., Foulkes R. (2025). IRL201104, A Novel Immunomodulatory Peptide, Shows Efficacy In An Allergen Driven Model Of Atopic Dermatitis. J. Allergy Clin. Immunol..

[104] (2021). A Phase 2a, Double-Blind, Placebo-Controlled, Multi-Center Study to Assess the Efficacy, Safety, and Tolerability of IRL201104 in Adult Participants with Active Eosinophilic Esophagitis (EoE).

[105] (2024). A Multicentre, SAD, and MAD Clinical Trial to Evaluate the Safety, Tolerability, Pharmacokinetics, and Pharmacodynamics of IV Treatment of CALY-002 in Healthy Subjects and Subjects with Celiac Disease and Eosinophilic Esophagitis (CALY-CL19-001).

[106] Cartier A., Hla T. (2019). Sphingosine 1-Phosphate: Lipid Signaling in Pathology and Therapy. Science.

[107] Dellon E.S., Collins M.H., Bredenoord A.J., Philpott H., Biedermann L., Dulcine M., Nguyen-Cleary T., Su C., Yu J., Tan H. (2025). Etrasimod as a Treatment for Eosinophilic Oesophagitis (VOYAGE): A Double-Blind, Placebo-Controlled, Randomised, Phase 2 Trial. Lancet Gastroenterol. Hepatol..

[108] Arna-20211231. https://www.sec.gov/Archives/edgar/data/1080709/000108070922000006/arna-20211231.htm.

[109] Pfizer Inc Pfizer Pipeline. Q1 2025 Pipeline Update (Programs Discontinued from Development Since February 4, 2025). Pfizer Inc. [Internet]. https://cdn.pfizer.com/pfizercom/product-pipeline/Q1_2025_Pipeline_Update_29APR2025.pdf?VersionId=7hjVCBiYrHhTyTEE1tgA5DD8Hp4qB0vo.

[110] Alvarado D., Maurer M., Gedrich R., Seibel S.B., Murphy M.B., Crew L., Goldstein J., Crocker A., Vitale L.A., Morani P.A. (2022). Anti-KIT Monoclonal Antibody CDX-0159 Induces Profound and Durable Mast Cell Suppression in a Healthy Volunteer Study. Allergy.

[111] Alvarado D., Lu Y., Shoda T., Caldwell J.M., Keler T., Rothenberg M.E. (2023). Strong Association of Mast Cells with Eosinophilic Esophagitis-Specific Signatures. Allergy.

[112] (2025). A Phase 2, Randomized, Double-Blind, Placebo-Controlled Study to Assess the Efficacy and Safety of Barzolvolimab (CDX-0159) in Adults with Active Eosinophilic Esophagitis (The “EvolvE” Study).

[113] Alvarado D., Bloom B.J., Meltzer-Podolske M.C., Crowley E., Rogalski M., Young D., Dellon E.S. (2025). Mo1345: Intraepithelial Mast Cells Are Elevated in Active Eosinophilic Esophagitis and Correlate with Eosinophils: Baseline Data from a Randomized Controlled Trial of Barzolvolimab. Gastroenterology.

[114] Azouz N.P., Klingler A.M., Pathre P., Besse J.A., Baruch-Morgenstern N.B., Ballaban A.Y., Osswald G.A., Brusilovsky M., Habel J.E., Caldwell J.M. (2020). Functional Role of Kallikrein 5 and Proteinase-Activated Receptor 2 in Eosinophilic Esophagitis. Sci. Transl. Med..

[115] (2025). An Open-Label Study of Zemaira (Alpha 1-Trypsin Inhibitor) in Subjects with Eosinophilic Esophagitis (Zemaira Eosinophilic Esophagitis Pilot Study–ZEEPS).

[116] Dellon E.S. (2024). Eosinophilic Esophagitis: What’s in a Name?. Dig. Dis. Sci..

[117] Bertin L., Savarino E.V. (2025). Editorial: Targeting the Future of Eosinophilic Oesophagitis Management. Aliment. Pharmacol. Ther..

[118] Greuter T., Straumann A., Fernandez-Marrero Y., Germic N., Hosseini A., Yousefi S., Simon D., Collins M.H., Bussmann C., Chehade M. (2022). Characterization of Eosinophilic Esophagitis Variants by Clinical, Histological, and Molecular Analyses: A Cross-Sectional Multi-Center Study. Allergy.

[119] Underwood B., Troutman T.D., Schwartz J.T. (2023). Breaking down the Complex Pathophysiology of Eosinophilic Esophagitis. Ann. Allergy Asthma Immunol..

[120] Beveridge C.A., Hermanns C., Thanawala S., Chatterjee A., Sharma N., Vura N.V.R.K., Yang Q., Qin Y., Thota P., Hoscheit M. (2025). Predictors of Persistent Symptoms in Eosinophilic Esophagitis after Remission: Fibrostenosis, Eosinophilia, Anxiety, and Depression. Dis. Esophagus.

[121] Center for Drug Evaluation and Research Eosinophilic Esophagitis: Developing Drugs for Treatment Guidance for Industry. https://www.fda.gov/regulatory-information/search-fda-guidance-documents/eosinophilic-esophagitis-developing-drugs-treatment-guidance-industry.

[122] Ruffner M.A., Cianferoni A. (2020). Phenotypes and Endotypes in Eosinophilic Esophagitis. Ann. Allergy Asthma Immunol..

[123] Shoda T., Wen T., Aceves S.S., Abonia J.P., Atkins D., Bonis P.A., Caldwell J.M., Capocelli K.E., Carpenter C.L., Collins M.H. (2018). Eosinophilic Oesophagitis Endotype Classification by Molecular, Clinical, and Histopathological Analyses: A Cross-Sectional Study. Lancet Gastroenterol. Hepatol..

[124] Wechsler J.B., Bolton S.M., Amsden K., Wershil B.K., Hirano I., Kagalwalla A.F. (2018). Eosinophilic Esophagitis Reference Score Accurately Identifies Disease Activity and Treatment Effects in Children. Clin. Gastroenterol. Hepatol..

[125] Araujo I.K., Shehata C., Hirano I., Gonsalves N., Kahrilas P.J., Tetreault M.-P., Schauer J.M., Farina D., Peterson S., Kou W. (2024). The Severity of Reduced Esophageal Distensibility Parallels Eosinophilic Esophagitis Disease Duration. Clin. Gastroenterol. Hepatol..

[126] Carlson D.A., Hirano I., Gonsalves N., Kahrilas P.J., Araujo I.K., Yang M., Tetreault M.-P., Pandolfino J.E. (2024). Composite Score of Physiomechanical Esophageal Function Using Functional Lumen Imaging Probe Panometry in Eosinophilic Esophagitis. Gastrointest. Endosc..

[127] Carlson D.A., Hirano I., Gonsalves N., Kahrilas P.J., Araujo I.K., Yang M., Tetreault M.-P., Pandolfino J.E. (2023). A PhysioMechanical Model of Esophageal Function in Eosinophilic Esophagitis. Gastroenterology.

[128] Visaggi P., Del Corso G., Solinas I., Ovidi F., Adamo G., Dulmin I., Baiano Svizzero F., Bellini M., Savarino E.V., de Bortoli N. (2024). Adaptive Behaviors, Esophageal Anxiety, and Hypervigilance Modify the Association Between Dysphagia Perception and Histological Disease Activity in Eosinophilic Esophagitis. Am. J. Gastroenterol..

[129] de Rooij W.E., Evertsz F.B., Lei A., Bredenoord A.J. (2022). General Well-Being and Coping Strategies in Adult Eosinophilic Esophagitis Patients. J. Neurogastroenterol. Motil..

[130] de Rooij W.E., Bennebroek Evertsz’ F., Lei A., Bredenoord A.J. (2021). Mental Distress among Adult Patients with Eosinophilic Esophagitis. Neurogastroenterol. Motil..

[131] Sorge A., Aldinio G., Marinoni B., Visaggi P., Penagini R., Maniero D., Ghisa M., Marabotto E., de Bortoli N., Pasta A. (2025). Distribution of Esophageal Inflammation in Patients with Eosinophilic Esophagitis and Its Impact on Diagnosis and Outcome. Dig. Liver Dis..

[132] Bell J., Cook S., Edwards T.L., Rice T.W., Self W.H., Wheeler A., Rhoads J., Stewart T.G., Pulley J.M., Benhoff K. (2022). Using a Multicultural and Multilingual Awareness-Raising Strategy to Enhance Enrollment of Racially Underrepresented Minoritized Communities—The PassITON Trial. J. Clin. Transl. Sci..

[133] Chang J.W., Chen V.L., Rubenstein J.H., Dellon E.S., Wallner L.P., De Vries R. (2022). What Patients with Eosinophilic Esophagitis May Not Share with Their Providers: A Qualitative Assessment of Online Health Communities. Dis. Esophagus.

[134] Kewalramani A., Waddell J., Puppa E.L. (2021). Telemedicine during the Coronavirus Disease 2019 Pandemic for Pediatric Patients with Eosinophilic Esophagitis. Ann. Allergy. Asthma. Immunol..

[135] van Rhijn B.D., Warners M.J., Curvers W.L., van Lent A.U., Bekkali N.L., Takkenberg R.B., Kloek J.J., Bergman J.J.G.H.M., Fockens P., Bredenoord A.J. (2014). Evaluating the Endoscopic Reference Score for Eosinophilic Esophagitis: Moderate to Substantial Intra- and Interobserver Reliability. Endoscopy.

[136] Rivas A., Ahmed N.S., Yuan Y., Qasim A., O’Gorman D.B., Feagan B.G., Jairath V., Bredenoord A.J., Dellon E.S., Ma C. (2025). Meta-Analysis: Evaluating Placebo Rates Across Outcomes in Eosinophilic Oesophagitis Randomised Controlled Trials. Aliment. Pharmacol. Ther..

[137] Enck P., Klosterhalfen S. (2019). Placebos and the Placebo Effect in Drug Trials. Handb. Exp. Pharmacol..

[138] Muir A., Moore H., Spergel J. (2019). To Treat or Not to Treat: The Minimally Symptomatic EoE Patient. Ann. Allergy Asthma Immunol..

[139] Millum J., Grady C. (2013). The Ethics of Placebo-Controlled Trials: Methodological Justifications. Contemp. Clin. Trials.

[140] Abe Y., Kikuchi R., Sasaki Y., Mizumoto N., Yagi M., Onozato Y., Watabe T., Goto H., Miura T., Sato R. (2024). Long-Term Course of Untreated Asymptomatic Esophageal Eosinophilia and Minimally Symptomatic Eosinophilic Esophagitis. Endosc. Int. Open.

[141] Ben-Eltriki M., Rafiq A., Paul A., Prabhu D., Afolabi M.O.S., Baslhaw R., Neilson C.J., Driedger M., Mahmud S.M., Lacaze-Masmonteil T. (2024). Adaptive Designs in Clinical Trials: A Systematic Review-Part I. BMC Med. Res. Methodol..

[142] Muehlemann N., Zhou T., Mukherjee R., Hossain M.I., Roychoudhury S., Russek-Cohen E. (2023). A Tutorial on Modern Bayesian Methods in Clinical Trials. Ther. Innov. Regul. Sci..

[143] Choodari-Oskooei B., Blenkinsop A., Handley K., Pinkney T., Parmar M.K.B. (2024). Multi-Arm Multi-Stage (MAMS) Randomised Selection Designs: Impact of Treatment Selection Rules on the Operating Characteristics. BMC Med. Res. Methodol..

[144] Djulbegovic B., Kumar A., Glasziou P.P., Perera R., Reljic T., Dent L., Raftery J., Johansen M., Di Tanna G.L., Miladinovic B. (2012). New Treatments Compared to Established Treatments in Randomized Trials. Cochrane Database Syst. Rev..

[145] Clemmensen P., Holmvang L., Grande P., Wagner G.S. (1999). “Add-on” Research in Clinical Trials: Are We Asking the Right Questions?. J. Electrocardiol..

[146] Sedgwick P. (2014). What Is a Crossover Trial?. BMJ.

[147] Taft T.H., Carlson D.A., Simons M., Zavala S., Hirano I., Gonsalves N., Pandolfino J.E. (2021). Esophageal Hypervigilance and Symptom-Specific Anxiety in Patients with Eosinophilic Esophagitis. Gastroenterology.

[148] Mirmiran P., Bahadoran Z., Gaeini Z. (2021). Common Limitations and Challenges of Dietary Clinical Trials for Translation into Clinical Practices. Int. J. Endocrinol. Metab..

[149] Tien D.S., Hockey M., So D., Stanford J., Clarke E.D., Collins C.E., Staudacher H.M. (2024). Recommendations for Designing, Conducting, and Reporting Feeding Trials in Nutrition Research. Adv. Nutr..

[150] Fink M., Simons M., Tomasino K., Pandit A., Taft T. (2022). When Is Patient Behavior Indicative of Avoidant Restrictive Food Intake Disorder (ARFID) Vs Reasonable Response to Digestive Disease?. Clin. Gastroenterol. Hepatol..

[151] Fonseca N.K.O., Curtarelli V.D., Bertoletti J., Azevedo K., Cardinal T.M., Moreira J.D., Antunes L.C. (2024). Avoidant Restrictive Food Intake Disorder: Recent Advances in Neurobiology and Treatment. J. Eat. Disord..

[152] Visaggi P., Mariani L., Pardi V., Rosi E.M., Pugno C., Bellini M., Zingone F., Ghisa M., Marabotto E., Giannini E.G. (2021). Dietary Management of Eosinophilic Esophagitis: Tailoring the Approach. Nutrients.

[153] Ketchem C.J., Starling A.S. (2025). Insights into the Natural History and Disease Course of Eosinophilic Esophagitis. Ann. Allergy Asthma Immunol..

[154] Chang N.C., Thakkar K.P., Ketchem C.J., Eluri S., Reed C.C., Dellon E.S. (2022). A Gap in Care Leads to Progression of Fibrosis in Eosinophilic Esophagitis Patients. Clin. Gastroenterol. Hepatol..

[155] Runge T.M., Eluri S., Woosley J.T., Shaheen N.J., Dellon E.S. (2017). Control of Inflammation Decreases the Need for Subsequent Esophageal Dilation in Patients with Eosinophilic Esophagitis. Dis. Esophagus.

[156] Knezevic N.N., Sic A., Worobey S., Knezevic E. (2025). Justice for Placebo: Placebo Effect in Clinical Trials and Everyday Practice. Medicines.

[157] Dellon E.S., Savarino E.V., Zaghloul S., Angello J.T., Zhang M., Raphael B.P., Radwan A., Bredenoord A.J. (2025). Study Design of the Phase IV, Randomized, Placebo-Controlled REMOdeling with Dupilumab in Eosinophilic Esophagitis Long-Term (REMODEL) Trial. Ther. Adv. Gastroenterol..

[158] Hirano I., Furuta G.T. (2020). Approaches and Challenges to Management of Pediatric and Adult Patients with Eosinophilic Esophagitis. Gastroenterology.

[159] Oliva S., Arrigo S., Bramuzzo M., Cisarò F., Dabizzi E., Di Nardo G., Gandullia P., Martinelli M., Mennini M., Monica F. (2025). Eosinophilic Esophagitis in Children and Adolescents: A Clinical Practice Guideline. Ital. J. Pediatr..

[160] Główczewski A., Krogulska A. (2022). Formulations of Topical Steroids in Eosinophilic Esophagitis-Current Treatment and Emerging Possibilities. J. Clin. Med..

[161] Mehta P., Pan Z., Zhou W., Kwan B.M., Furuta G.T. (2023). Medication Adherence Rates in Adolescents with Eosinophilic Esophagitis Are Low and Are Associated with Health Habits. J. Pediatr. Gastroenterol. Nutr..

[162] Brokmann F., Simonek P., Rosenbaum C. (2024). Modification of the Biorelevant Release Testing of Esophageal Applied Mucoadhesive Films and Development of Formulation Strategies to Increase the Mucosal Contact Time. Pharmaceutics.

[163] Prasher A., Shrivastava R., Dahl D., Sharma-Huynh P., Maturavongsadit P., Pridgen T., Schorzman A., Zamboni W., Ban J., Blikslager A. (2021). Steroid Eluting Esophageal-Targeted Drug Delivery Devices for Treatment of Eosinophilic Esophagitis. Polymers.

[164] Lim A.W., Talley N.J., Walker M.M., Storm G., Hua S. (2023). Current Status and Advances in Esophageal Drug Delivery Technology: Influence of Physiological, Pathophysiological and Pharmaceutical Factors. Drug Deliv..

[165] Spennacchio A., Lopalco A., Racaniello G.F., Cutrignelli A., la Forgia F.M., Fontana S., Cristofori F., Francavilla R., Lopedota A.A., Denora N. (2024). Mucoadhesive Budesonide Solution for the Treatment of Pediatric Eosinophilic Esophagitis. Pharmaceuticals.

[166] Mennini M., Tambucci R., Riccardi C., Rea F., De Angelis P., Fiocchi A., Assa’ad A. (2021). Eosinophilic Esophagitis and Microbiota: State of the Art. Front. Immunol..

[167] Sherrill J.D., Rothenberg M.E. (2014). Genetic and Epigenetic Underpinnings of Eosinophilic Esophagitis. Gastroenterol. Clin. N. Am..

[168] Noble S.-L., Tyrrell R., Mules T.C., Inns S. (2025). Non-Invasive Biomarkers to Diagnose and Monitor Eosinophilic Esophagitis: A Systematic Review. Front. Med..

[169] Dellon E.S., Higgins L.L., Beitia R., Rusin S., Woosley J.T., Veerappan R., Selitsky S.R., Parker J.S., Genta R.M., Lash R.H. (2016). Prospective Assessment of Serum Periostin as a Biomarker for Diagnosis and Monitoring of Eosinophilic Esophagitis. Aliment. Pharmacol. Ther..

[170] Buendia M.A., Choksi Y.A., Hiremath G. (2022). Relapse of Eosinophilic Esophagitis on Dupilumab. JPGN Rep..

[171] Visaggi P., Del Corso G., Baiano Svizzero F., Ghisa M., Bardelli S., Venturini A., Stefani Donati D., Barberio B., Marciano E., Bellini M. (2024). Artificial Intelligence Tools for the Diagnosis of Eosinophilic Esophagitis in Adults Reporting Dysphagia: Development, External Validation, and Software Creation for Point-of-Care Use. J. Allergy Clin. Immunol. Pract..

[172] Xu X., Chen H., Chen Y., Fan L. (2025). Application of Artificial Intelligence in Eosinophilic Esophagitis. Front. Immunol..

